# A vein wall cell atlas of murine venous thrombosis determined by single-cell RNA sequencing

**DOI:** 10.1038/s42003-023-04492-z

**Published:** 2023-01-31

**Authors:** Elise DeRoo, Ting Zhou, Huan Yang, Amelia Stranz, Peter Henke, Bo Liu

**Affiliations:** 1 Department of Surgery, School of Medicine and Public Health, University of Wisconsin-Madison, Madison, WI 53705 USA; 2 Department of Surgery, Division of Vascular Surgery, University of Michigan, Ann Arbor, MI 48109 USA; 3 Department of Cellular and Regenerative Biology, School of Medicine and Public Health, University of Wisconsin-Madison, Madison, WI 53705 USA

**Keywords:** Thrombosis, Thrombosis

## Abstract

Deep vein thrombosis (DVT) is a common clinical problem, but its cellular and molecular mechanisms remain incompletely understood. In this study, we performed single-cell RNA sequencing on mouse inferior vena cava (IVC) 24 h after thrombus-inducing IVC ligation or sham operation. 9 cell types composed of multiple subpopulations were identified. Notable transcriptomic changes induced by DVT included a marked inflammatory response, elevated hypoxia, and globally reduced myogenesis. Analysis of individual cell populations revealed increased inflammation and reduced extracellular matrix production across smooth muscle cells and fibroblasts, juxtaposed against an early phenotypic shift in smooth muscle cell populations away from a contractile state. By characterizing the transcriptomic changes in the vein wall during acute venous thrombosis at the single-cell level, this work provides novel insights into early pathological events in the vein wall that may potentiate thrombus formation and result in long term adverse venous remodeling.

## Abstract (web summary)

Early changes in smooth muscle cell populations in the vein wall following deep vein thrombosis in mouse may potentiate thrombus formation and venous remodeling.

## Introduction

Venous thromboembolism, which encompasses both deep vein thrombosis (DVT) and pulmonary embolism, is a common disease, with an estimated incidence of 300,000–600,000 events in the United States annually^1^. While pulmonary embolism is associated with high mortality, DVT is associated with substantial morbidity, with 30–50% of patients going on to experience post-thrombotic syndrome^2,3^. As venous thromboembolism is a leading cause of disability-adjusted life years^4^, a better understanding of this disease and its molecular mechanisms is necessary in order to develop targeted treatments and preventative strategies.

Venous thrombogenesis is thought to involve vein wall activation followed by inflammatory cell and platelet accumulation, which further promote a pro-coagulant, pro-inflammatory local environment^5–11^. The precise roles of each vascular and immune cell population in thrombus formation, resolution, and post-thrombotic vein wall remodeling are incompletely understood. Studies using bulk RNA sequencing to examine early transcriptomic changes in the vein wall across murine^12^ and porcine^13^ models of DVT have been performed. However, the molecular signature of DVT within the vein wall at single-cell resolution is still unknown.

Open surgery for DVT in human is rarely performed, and when done, it typically involves thrombectomy alone without vein removal. To identify the cell populations present in the vein wall and their response in the setting of DVT, we performed single-cell RNA sequencing (scRNA-seq) on the inferior vena cava (IVC) of mice that underwent IVC ligation or sham surgery. Our analysis provides a comprehensive, unbiased, and high-resolution view of the cellular and molecular changes in the DVT-bearing vein wall, and will be paramount in designing future evidence-based, hypothesis-driven studies in the field of DVT research.

## Results

### Identification of vein wall cell populations in murine DVT

**Fig. 1:**
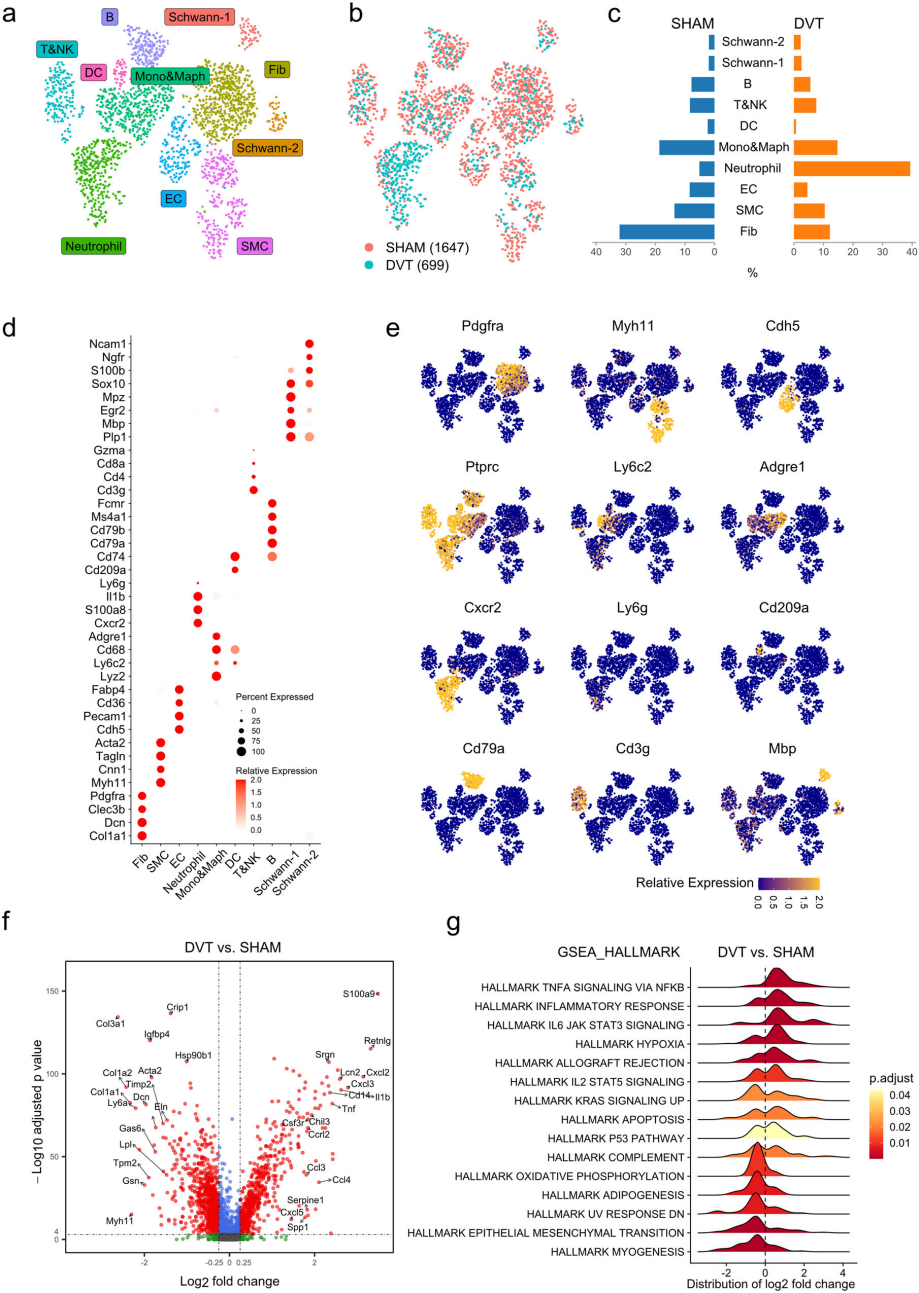
Identification of cell populations in mouse deep vein thrombosis (DVT) model. 8 to 12-week-old male C57BL6/J mice were subjected to the inferior vena cava (IVC) ligation model of DVT. IVC was collected 24 h after ligation. IVC from 8 sham or 5 DVT-bearing mice was pooled as one sample in each group. **a** Uniform manifold approximation and projection (UMAP) plot of cell clusters presented in mouse IVCs (sham and DVT data combined). **b** Cell distributions in sham and DVT groups. **c** Percentages of cell populations in sham and DVT groups. **d** Expression of canonical cell markers in each cluster. **e** UMAP plots of cell markers in sham and DVT data combined. **f** Volcano plot of differentially expressed genes (DEGs) in DVT versus sham group. **g** Gene Set Enrichment Analysis (GSEA) of up- and down- regulated gene sets in DVT versus sham group. Hallmark gene sets were used.

Twenty-four hours after surgery, IVC-ligated mice developed similarly sized thrombi (length 9.2±0.6 mm, *n* = 8 mice in sham group, *n* = 5 mice in DVT group), while no thrombi were observed in the sham group (Supplementary Fig. 1a). Single-cell suspensions were prepared from sham and DVT-associated IVCs. Tissue from 8 sham or 5 DVT-bearing mice was pooled as one sample in each group. A total of 2343 viable cells, including 1644 cells in the sham group and 699 cells in the DVT group, were recovered after removing low-quality cells. Unsupervised Seurat-based clustering identified 9 distinct cell populations that were present in the vein wall (smooth muscle cells [SMCs], fibroblasts [Fibs], endothelial cells [ECs], neutrophils, monocytes/macrophages [Mono&Maphs], T&NK cells, B cells, dendritic cells [DCs], & Schwann cells) (Fig. 1a–c, Supplementary Data 7). Biological identities were assigned based on gene expression patterns of established canonical markers of each cell type^14–19^ (Fig. 1d, e, and Supplementary Fig. 1b, Supplementary Data 7). IVC ligation changed the proportions of cell populations. Compared to the sham condition, IVCs in the DVT group contained a higher percentage of neutrophils, and lower percentages of fibroblasts, SMCs, ECs, Mono&Maphs, T&NK cells, and B cells (Fig. 1c, Supplementary Data 7).

### Major changes in transcriptomes and biological processes in murine DVT-bearing vein wall

Differential expression analysis revealed over 1,757 genes that were altered by DVT (Fig. 1f and Supplementary Data 1). As shown in Supplementary Fig. 1c and 2a, the most upregulated genes included numerous pro-inflammatory genes, and the most downregulated genes consisted of many genes encoding extracellular matrix (ECM) proteins and regulators. Gene set enrichment analysis (GSEA) showed that inflammatory responses were the most upregulated biological processes. Gene sets such as hypoxia and apoptosis showed mixed up and downregulation post-DVT. Indeed, while GSEA revealed both up and downregulation of apoptosis gene set in DVT, greater burden of genes mediating apoptosis were upregulated. TUNEL staining confirmed higher number of apoptotic cells within the vein wall of DVT-bearing mice (Fig. 1g, Supplementary Fig. 2b). Downregulated gene sets were related to myogenesis, epithelial-mesenchymal transition, and oxidative phosphorylation. (Fig. 1g and Supplementary Fig. 3a). Comparison among differentially expressed genes (DEGs) from our dataset, bulk RNA sequencing data of an acute porcine femoral vein stasis model of DVT^13^, and a data set of inferred gene changes in human with venous thrombosis^20^ revealed 55 common DEGs, including genes related to inflammation, hypoxia, and cell death (Supplementary Fig. 3b).

### Cell population analysis

#### Smooth muscle cells

**Fig. 2:**
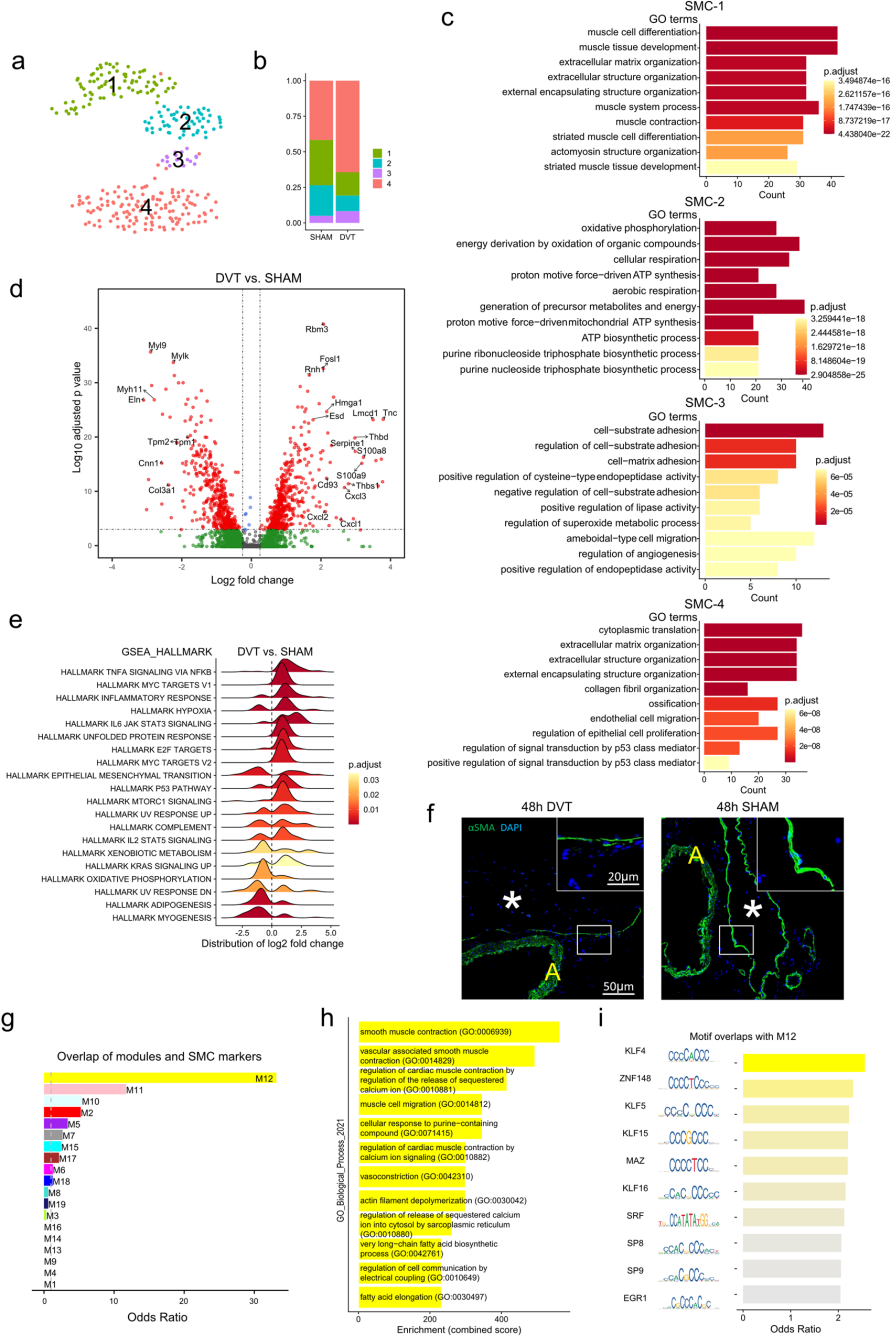
Gene expression heterogeneity of smooth muscle cells (SMCs). **a** UMAP plot of sub-populations in SMCs of sham and DVT data combined. **b** Relative distribution of sub-populations in sham and DVT groups. **c** Gene Ontology (GO) analysis of each SMC sub-population in comparison to the other three sub-populations. **d** Volcano plot of DEGs in total SMCs (DVT vs sham group). **e** GSEA of the altered gene sets in DVT versus sham group. **f** Immunostaining of smooth muscle cell marker α-smooth muscle actin (α-SMA) within IVC or IVC/thrombus cross-sections 48 h after sham surgery or IVC ligation (DVT group). DAPI was used to stain nuclei. Vessel lumen or thrombus depicted with asterisk. Yellow “A” indicates aorta. Area encompassed by white dotted box is shown magnified in the insert. *N* = 3 mice for each group. **g** Association between SMC markers and each co-expression module. **h** Enriched biological processes in module 12. **i** Association between transcription factor binding motifs and module 12.

Four subpopulations of SMCs were identified in both sham and DVT-IVCs (Fig. 2a). Relative decreases in SMC-1 and 2 were noted in the DVT condition, while SMC-3 and 4 were expanded (Fig. 2b and Supplementary Fig. 4a, Supplementary Data 7). Gene enrichment and pathway analysis showed that SMC-1 highly expressed genes controlling cytoskeletal structure and muscle contraction, suggesting a contractile phenotype. SMC-2 showed enrichment of genes involved in oxidative phosphorylation and cellular respiration, indicating they were metabolically active. SMC-3 was enriched in genes regulating cell-matrix adhesion, likely responsive to biomechanical stress. Finally, SMC-4, the most expanded population, was characterized by high expression of genes controlling ECM organization and translation machinery, suggesting a synthetic phenotype (Fig. 2c and Supplementary Fig. 4b, Supplementary Data 7).

Differential expression analysis revealed 579 upregulated and 494 downregulated genes in SMCs in the setting of DVT (Fig. 2d, Supplementary Fig. 4c, and Supplementary Data 2). Notable upregulated genes included drivers of inflammation (*S100a8, Cxcl3*), genes related to biomechanical stress, coagulation cascade (*Serpine1, Thbd*), and regulators of cell death/apoptosis (*Cd93*). Genes related to muscle contraction (*Myh11, Eln, Cnn1*) were downregulated. GSEA revealed increased expression of gene sets related to inflammatory response, hypoxia, and unfolded protein response in the DVT condition. In contrast, DVT decreased expression of gene sets related to adipogenesis and myogenesis (Fig. 2e and Supplementary Fig. 4d). As for the subpopulations, differential gene expression analysis identified that DVT altered the expression of 15 genes in the SMC-1 population compared with sham, while DVT did not significantly alter gene expression in the other SMC subpopulations (Supplementary Fig. 5). Immunostaining for α-smooth muscle actin (α-SMA) across vein wall specimens from DVT or sham-treated mice confirmed reduced α-SMA levels in the DVT-adjacent IVC (Fig. 2f). The subpopulations were validated by co-staining for the SMC marker, smooth muscle myosin heavy chain 11 (MYH11), and for the marker genes which were highly and uniquely expressed in each subpopulation (Fibronectin-1 [FN1] for SMC-1, Krüppel-like factor 2 [KLF2] for SMC-2, CD36 for SMC-3, and actin alpha cardiac muscle 1 [ACTC1] for SMC-4) (Supplementary Fig. 6a–d).

We further performed weighted gene co-expression network analysis (WGCNA) in SMCs^21^. 19 co-expressed gene modules were identified in SMCs from both sham and DVT groups (Supplementary Fig. 6e, f), with module 12 (M12) having the greatest overlap with SMC markers (Fig. 2g, Supplementary Data 7). Pathway analysis revealed that SMC contraction was the most enriched biological process in M12 (Fig. 2h, Supplementary Data 7). Assessing the effect of transcription factors on the expression of M12 genes indicated Krüppel-like factor 4 (KLF4) displayed the strongest association with the expression of genes in M12 (Fig. 2i and Supplementary Fig. 6g, Supplementary Data 7). Expression of these associated transcription factors varied across SMC subpopulations between sham and DVT conditions (Supplementary Fig. 7), as did M12 hub gene expression (Supplementary Fig. 8).

#### Fibroblasts

**Fig. 3:**
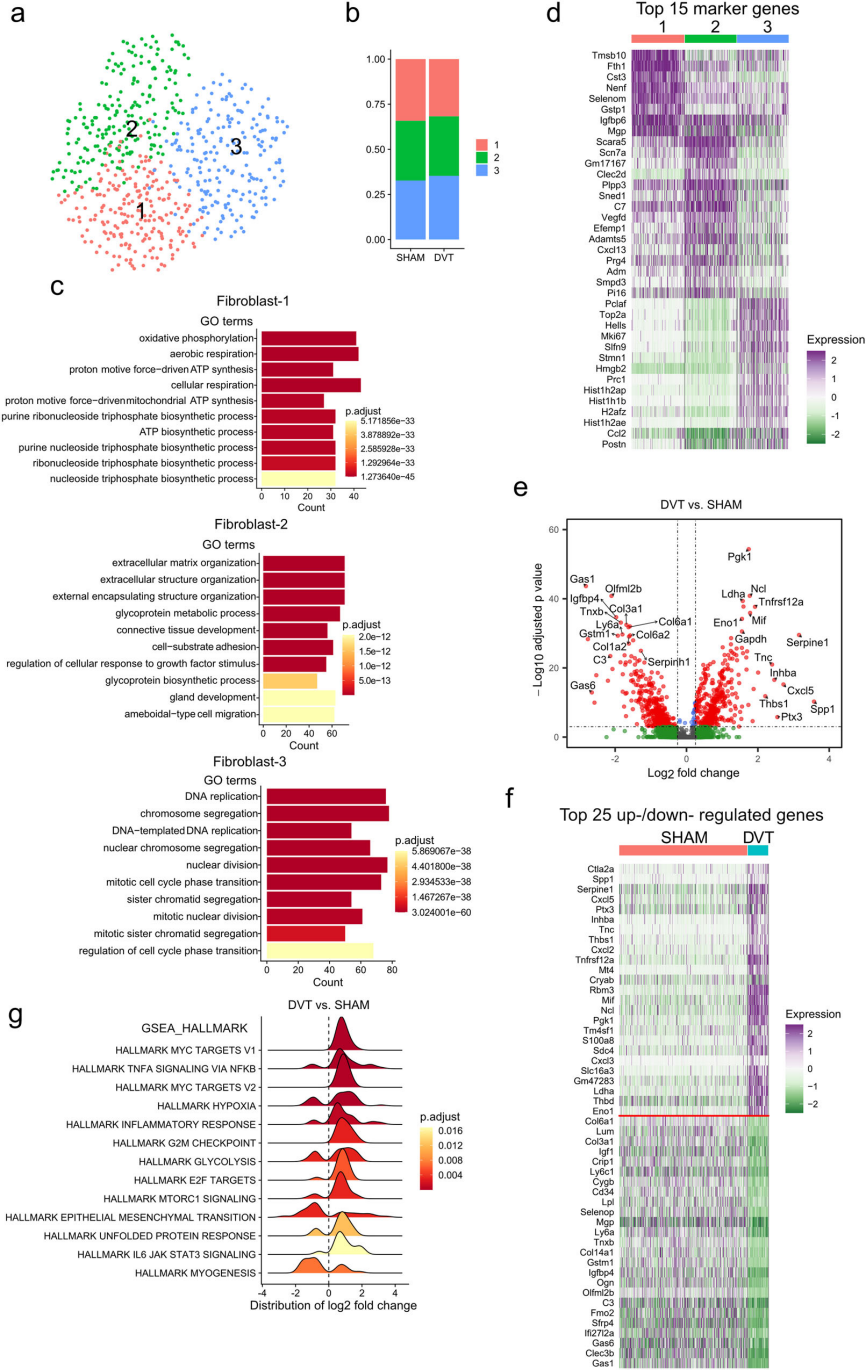
Gene expression heterogeneity of fibroblasts. **a** UMAP plot of sub-populations in fibroblasts of sham and DVT data combined. **b** Relative distribution of sub-populations in sham and DVT groups. **c** GO analysis of each fibroblast sub-population in comparison to the other two sub-populations. **d** Heatmap of the top 15 enriched genes in each fibroblast subpopulation in comparison to the other two sub-populations. **e** Volcano plot of DEGs in total fibroblasts (DVT vs sham group). **f** Heatmap of the top 25 up- and downregulated genes in DVT compared to sham group. Up- and downregulated genes were separate by a red line. **g** GSEA of the altered gene sets in DVT versus sham group.

Three subpopulations of fibroblasts were identified in both the sham and DVT conditions (Fig. 3a). While fibroblast number was reduced in the DVT condition, validated by immunostaining of fibroblast marker COL1A1 (Supplementary Fig. 9a), the relative distribution of fibroblast subpopulations was not changed (Fig. 3b, Supplementary Data 7). Fib-1 enriched in genes regulating oxidative phosphorylation and cellular respiration, indicating a highly metabolically active subpopulation. Fib-2 highly expressed genes involving ECM organization and connective tissue development, suggesting a synthetic phenotype. Fib-3 was characterized by high expression of genes controlling DNA replication, chromosome segregation, and nuclear division, suggesting a proliferating cell population (Fig. 3c, d, Supplementary Data 7).

Differential expression analysis revealed 445 upregulated and 344 downregulated genes in the setting of DVT (Fig. 3e, f, and Supplementary Data 3). Genes controlling regulation of translation (*Rbm3, Ncl*), cellular metabolism (*Pgk1, Ldha*,), coagulation (*Serpine1, Thbd*), inflammation (*Tnfrs12a, Mif, Cxcl3*), and cell-cell/cell-matrix interactions (*Thbs1*) were among the top upregulated in the setting of DVT. Genes regulating cell growth (*Gas1, Igfbp4, Igf1*) and ECM production (*Col3a1, Col6a1, Col14a1, Lum*) were among the most heavily downregulated, in contrast. GSEA showed high expression of gene sets related to hypoxia and inflammatory response, and reduced expression of gene sets related to myogenesis in the setting of DVT (Fig. 3g, and Supplementary Fig. 9b).

#### Endothelial cells

Three sub-populations of ECs were identified in sham and DVT-IVCs (Supplementary Fig. 10a). While fewer ECs were present in the DVT condition (Fig. 1b, c, Supplementary Data 7), overall proportions of subpopulations did not substantially differ (Supplementary Fig. 10b). EC-1 enriched in genes regulating lipid and fatty acid metabolism and migration, EC-2 highly expressed genes regulating ECM organization, and EC-3 was likely lymphatic ECs (Supplementary Fig. 10c, d).

Differential expression analysis indicated 61 significantly upregulated and 18 downregulated genes in DVT-adjacent IVCs (Supplementary Fig. 10e, f, and Supplementary Data 4). Notably, upregulated genes were related to angiogenesis (*Angpt2, Ccn1, Thbs1*), cytoskeleton (*Tpm4, Actb, Cnn2*), and coagulation (*Plaur*). Downregulated genes involved ECM production (*Col3a1, Col1a1, Eln*) and vascular integrity (*Ptprb, App*) (Supplementary Fig. 10e, f). GSEA revealed gene sets related to hypoxia were upregulated in EC by DVT (Supplementary Fig. 10g, h).

#### Neutrophils

**Fig. 4:**
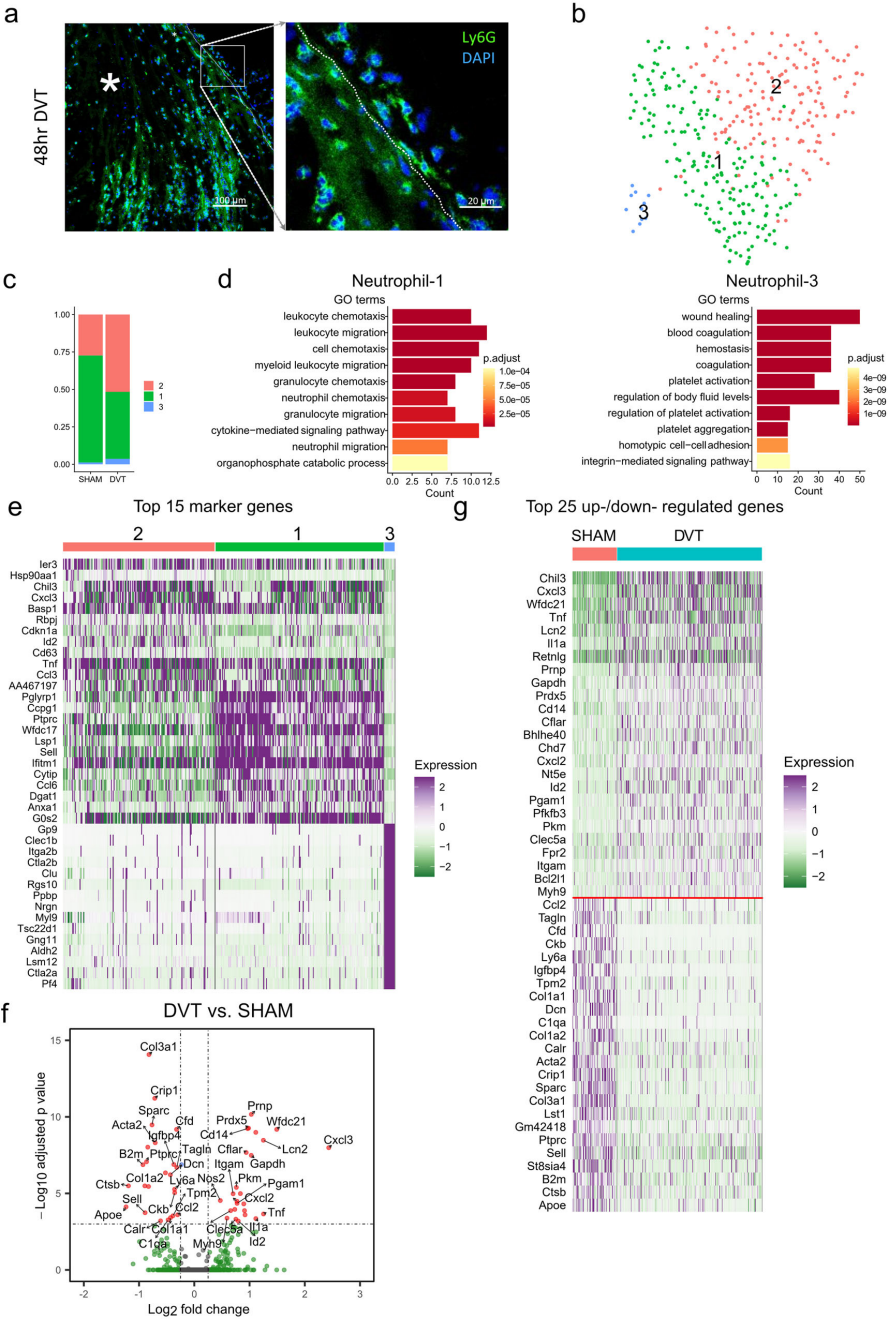
Gene expression heterogeneity of neutrophils. **a** Immunostaining of neutrophil marker Ly6G within IVC/thrombus cross-sections 48 h after IVC ligation (left panel). DAPI was used to stain nuclei. Thrombus depicted with asterisk. Vein wall/thrombus interface highlighted by white dotted line. Area encompassed by white box shown magnified in right panel. *N* = 3 mice for each group. **b** UMAP plot of sub-populations in neutrophils of sham and DVT data combined. **c** Relative distribution of sub-populations in sham and DVT groups. **d** GO analysis of Neutrophil-1 or Neutrophil-3 in comparison to the other two sub-populations. **e** Heatmap of the top 15 enriched genes in each sub-population in comparison to the other two sub-populations. **f** Volcano plot of DEGs in total neutrophils (DVT vs sham group). **g** Heatmap of the top 25 up- and downregulated genes in DVT compared to sham group. Up- and downregulated genes were separate by a red line.

Neutrophils became the predominant cell type within the vessel wall in DVT (Fig. 1b, c, Supplementary Data 7), a finding validated by Ly6G staining (Fig. 4a). Three subpopulations of neutrophils were identified. In the sham group, the majority of neutrophils belonged to Neutrophil-1. In contrast, DVT robustly expanded the neutrophil population where now Neutrophil-1 was reduced and Neutrophil-2 and −3 increased (Fig. 4b, c, Supplementary Data 7). Neutrophil-1 was characterized by high expression of genes related to chemotaxis and migration. Neutrophil-3 demonstrated enrichment of genes involved in regulating wound healing and coagulation. Gene Ontology analysis failed to reveal any individual upregulated gene sets in Neutrophil-2 (Fig. 4d, e, Supplementary Data 7).

Differential expression analysis revealed 26 upregulated and 24 downregulated genes in neutrophils in the DVT condition (Fig. 4f, g, and Supplementary Data 5). Notable upregulated genes included pro-inflammatory genes (*Cxcl3, Tnf, Il1a*), genes regulating oxidative stress (*Prdx5*), and cell death (*Lcn2, Cflar, Bcl2l1*). *Padi4*, which encodes the neutrophil extracellular trap (NET) driving enzyme PAD4, expression was increased in vein wall neutrophils by DVT, but this failed to achieve statistical significance. Significantly downregulated genes included a subset of those related to ECM production (*Col3a1, Col1a1, Sparc*), cytoskeletal structure (*Acta2, Tagln, Tmp2*), and the complement system (*Cfd, C1qa*).

#### Monocytes/Macrophages

**Fig. 5:**
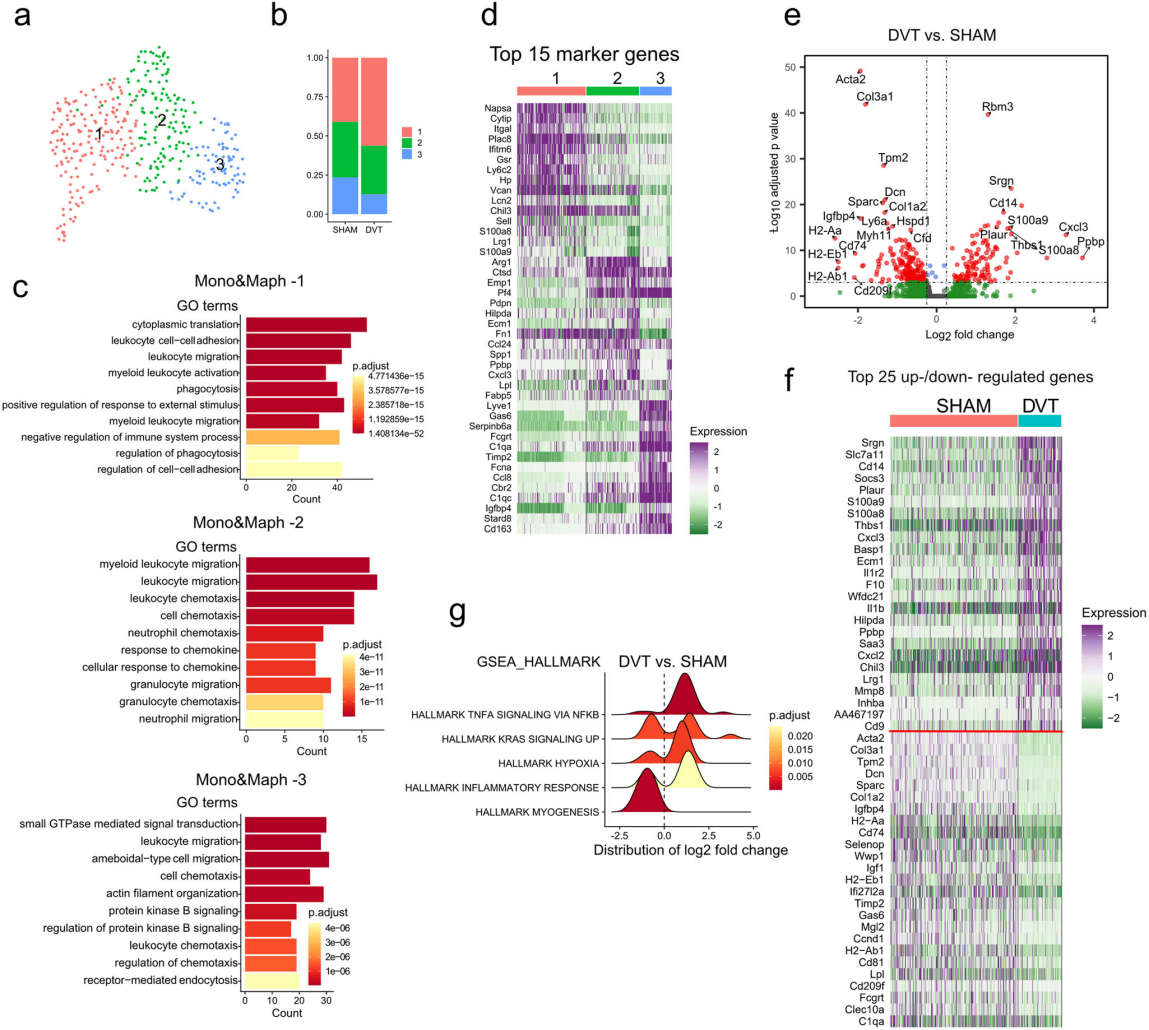
Gene expression heterogeneity of monocytes and macrophages. **a** UMAP plot of sub-populations in monocytes and macrophages of sham and DVT data combined. **b** Relative distribution of sub-populations in sham and DVT groups. **c** GO analysis of each sub-population in comparison to the other two sub-populations. **d** Heatmap of the top 15 enriched genes in each sub-population in comparison to the other two sub-populations. **e** Volcano plot of DEGs in total monocytes and macrophages (DVT vs sham group). **f** Heatmap of the top 25 up- and downregulated genes in DVT compared to sham group. Up- and downregulated genes were separate by a red line. **g** GSEA of the altered gene sets in DVT versus sham group.

Three monocyte/macrophage (Mono&Maph) populations were identified in both the sham and DVT conditions (Fig. 5a). DVT resulted in a relative increase in Mono&Maph-1 and decrease in Mono&Maph-3 (Fig. 5b, Supplementary Data 7). Mono&Maph-1 was characterized by high expression of genes related to leukocyte adhesion, activation, migration, and phagocytosis, while Mono&Maph-2 and Mono&Maph-3 highly expressed gene sets related to migration/chemotaxis and cytoskeletal organization (Fig. 5c, d, Supplementary Data 7).

Differential expression analysis revealed 115 upregulated and 194 downregulated genes in Mono&Maph in the setting of DVT (Fig. 5e, f, and Supplementary Data 6). Notable upregulated genes include those related to MMP production and processing (*Srgn, Mmp8*), immune response (*S100a8, S100a9, Cxcl3, Il1b*), thrombolysis (*Plaur*), coagulation (*F10*), and cell-matrix interactions (*Thbs1*). Notable downregulated genes include those related to the cytoskeleton (*Acta2, Myh11, Tpm2*), ECM (*Col1a1, Col3a1, Sparc*), and antigen presentation (*H2-Aa, H2-Ab1, Cd74*). GSEA revealed enrichment of gene sets related to TNFα-NFκB signaling and to a lesser extent hypoxia and inflammatory response, along with downregulation of gene sets related to myogenesis (Fig. 5g, and Supplementary Fig. 11).

#### Cell-cell communication

We sought to predict how different cell populations within the vein wall may communicate with one another using CellChat, an R toolkit that contains a database of 2,021 validated murine molecular interactions between signaling ligands, receptors, and their cofactors^22,23^.

**Fig. 6:**
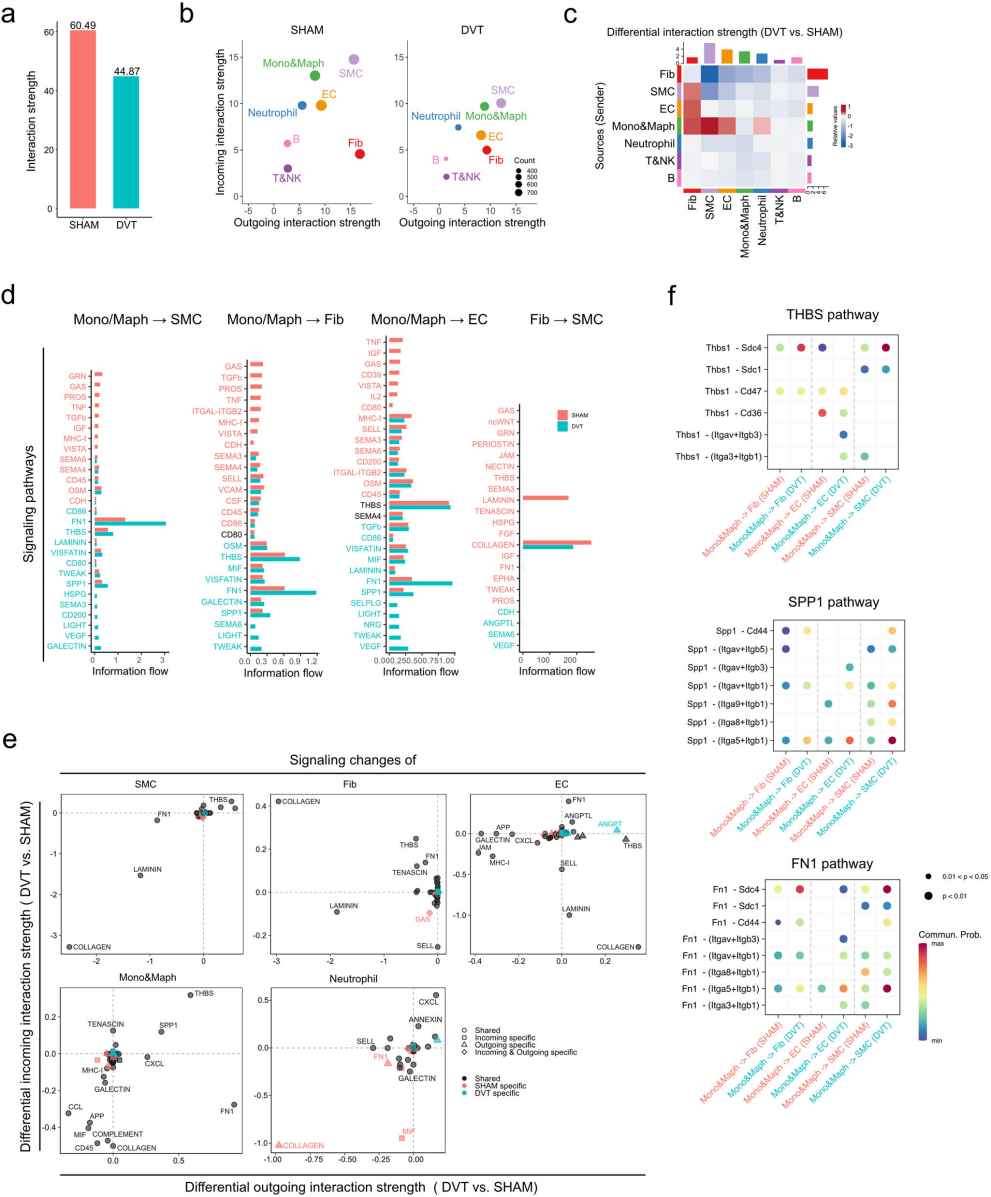
Inferred cell-cell communication network in mouse DVT. **a** Total interaction strength in sham and DVT groups. **b** Scatter plot of incoming and outgoing interaction strength of each cell population in sham and DVT groups. **c** Heatmap of differential interaction strength in DVT compared to the sham group. The top-colored bar plot represents the sum of column of values displayed in the heatmap (incoming signaling). The right-colored bar plot represents the sum of row of values (outgoing signaling). In the heatmap, red (or blue) represents increased (or decreased) signaling in DVT compared to sham group. Relative value = the communication probability from source to target in DVT group—the communication probability from source to target in sham group. **d** Overall information flow of each signaling pathway in sham and DVT groups. **e** Signaling changes of major cell types in DVT compared to sham group. **f** Bubble plot of the communication probability of all the significant ligand-receptor pairs that contributed to THBS, SPP1, and FN1 signaling sent from monocytes and macrophages to vascular cell types in sham and DVT groups.

Our analysis revealed greater intercellular communication probability, represented by interaction strength, in the sham group compared to the DVT group (Fig. 6a, Supplementary Data 7). SMCs and fibroblasts were the major signaling sources in the sham condition, and SMCs were the dominant signaling source in the DVT condition. SMCs and Mono&Maphs were the dominant signaling targets in both the sham and DVT conditions (Fig. 6b). Compared to the sham group, DVT increased signals sent from Mono&Maphs to SMCs, ECs, fibroblasts, and neutrophils. Signals from ECs to fibroblasts and from SMCs to fibroblasts were also increased in the DVT condition. In contrast, signals sent from fibroblasts to SMCs were decreased by DVT (Fig. 6c).

Given the enhancement of Mono&Maph signaling to SMC, fibroblast, and EC, and the decrease in fibroblast to SMC signaling in the DVT condition, a close examination of information flow between these cell populations was performed. FN1, THBS, and SPP1 signaling from Mono&Maph to SMC, fibroblast, and EC was enhanced in the DVT condition, while LAMININ and COLLAGEN signaling from fibroblast to SMC was reduced (Fig. 6d, Supplementary Data 7). An analysis of signaling changes within EC, SMC, fibroblast, neutrophil, and Mono&Maph was also performed (Fig. 6e). DVT induction altered THBS, ANGPTL/ANGPT, TENASCIN, and COLLAGEN signaling across many of the cell populations analyzed (Fig. 6e).

Analysis of outgoing FN1, THBS, and SPP signaling from Mono&Maph to vascular cells revealed several ligand-receptor pairs with high communication probabilities, including Fn1-Sdc4 and Fn1- (Itga5 + Itgb1), Thbs1-Sdc4, and Spp1- (Itga5 + Itgb1) respectively (Fig. 6f). A detailed analysis of gene expression within THBS, SPP1, and FN1 signaling pathways was performed across all vein wall cell populations in both the sham and DVT conditions (Supplementary Fig. 12).

## Discussion

With the power of scRNA-seq, we unbiasedly profiled the cellular and molecular signatures of the vein wall in a commonly used murine model of DVT. IVCs were examined 24 h after ligation, a time point when a thrombus is reliably formed and not firmly attached to the vein wall. The model utilized is consistent in producing similarly sized thrombi that have maximal contact with the vein wall^24^. Nine cell populations were identified in varying abundance in the sham and DVT-bearing IVC. The cellular heterogeneity of the IVC is further reflected by the unique gene expression patterns of subpopulations of each cell type.

Consistent with published studies^25–30^, our analysis demonstrated that neutrophil extravasation into the vein wall is a dominant cellular process in early DVT, top upregulated genes were mainly expressed by neutrophils and were related to inflammatory response. Indeed, studies have shown that neutrophils, along with monocytes and platelets, cooperate to promote venous thrombosis in mouse models of DVT^27,28,31,32^. While the role that pro-thrombotic NETs play within the environment of the thrombus is well established^27,33,34^, more broadly the role of neutrophils in venous thrombogenesis and vein wall remodeling appears to be complex. Mice deficient in NET formation (*Pad4*^*-/-*^*)* are protected from venous thrombosis in the IVC stenosis model of DVT^35^ and NETs can drive endothelial activation/death^34^, yet rats treated with a neutrophil-depleting antibody peri-IVC ligation develop larger thrombi and more adverse vein wall remodeling^36,37^. In this regard, our data provide an unbiased starting point to unravel the complex roles that neutrophils play in modulating post-thrombotic vein wall remodeling.

The bulk RNA sequencing study by Gromadziński et al.^13^ and the current scRNA-seq analysis identified a similar number of DEGs (1347 vs 1757). However, only 183 DEGs were shared by the two studies. The use of different animal species (castrated male pigs vs un-castrated male mice), different vein segments (femoral vein vs IVC), different ligation model (ligate both proximal and distal ends with thrombin administrated in the closed segment, vs ligate IVC only inferior to the left renal vain), and different sequencing technologies (bulk RNA sequencing vs scRNA-seq) are likely to account for the difference in DEGs. Among the many unique features of scRNA-seq, this more recent RNA sequencing technique enables identification of DEGs in each of the cell populations. Furthermore, identified DEGs can be appreciated within the unique transcriptomic environment of a cell type of interest.

Across all major vein wall cell populations, genes encoding ECM were downregulated in the DVT condition, and predominantly expressed by fibroblasts and SMCs. While this may in part be a consequence of acute inflammation and stand in contrast to the gene expression changes expected in the chronically injured post-thrombotic vein wall^38,39^, this analysis highlights that profound changes occur to the architecture of the vein wall ECM early in the course of DVT. Across multiple cell populations, gene sets involved in myogenesis were downregulated by DVT. The SMC-4 population (likely synthetic SMCs) expanded in the DVT condition at the expense of the SMC-1 population (likely contractile SMCs). The reduction in *Acta2*, *Myh11*, and *Cnn1* gene expression within SMC in the DVT condition confirms this early change in SMC phenotype. This change in the distribution of SMC subpopulations points to an unexpectedly early phenotypic change in the vein wall that may ultimately contribute to the development of post-thrombotic syndrome. Post-thrombotic syndrome is characterized by increased vessel wall stiffness, a phenotypic switch from a contractile to synthetic state among vascular SMCs, and heavy collagen deposition^3,38,40^. While fibrotic and fibroproliferative changes within the vessel wall have been documented at late timepoints in the course of DVT resolution^38^, never before has evidence of this phenotypic change been inferred at such an early timepoint.

KLF4 is a well-established transcription factor that promotes the transition of SMC from a contractile to a synthetic/mesenchymal like state in the context of arterial disease^41,42^. Analysis of gene expression patterns of the transcription factors regulating SMC-specific co-expressed gene module revealed no significant change in KLF4 expression between sham and DVT groups within SMC-4, but did show a dramatic increase in KLF16 and decrease in KLF15 expression within this subpopulation. Further research regarding the transcription factors that drive SMC phenotypic changes in DVT is merited. At the clinical level, our analysis highlights a potential need for hyper-acute interventions and therapies that can interrupt this adverse vein wall remodeling, which appears to begin from the moment of thrombus formation^43^.

Finally, our study provides novel insights into the complex cell-cell communication network unfolding in the thrombus-bearing vein wall. Despite neutrophils being the predominant leukocyte within the vein wall in acute DVT^38,44,45^, Mono&Maph dominated outgoing cell-cell communication to EC, SMC, and fibroblast. Communication via THBS, SPP1, and FN1 signaling pathways from Mono&Maph to other vascular cell types was uniformly increased in the DVT condition. The significance of these cell-cell communication pathways within the context of DVT is worthy of future investigation.

One limitation of scRNA-seq is its focus on the transcriptome. While we have confirmed a limited number of gene expression changes at the protein level by immunostaining, to validate every gene change at the protein level would be impractical. In addition, we predicted the intercellular communication network via CellChat^22,23^, validation of the inferred cell-cell communication would be highly informative. Another limitation of the current scRNA-seq technique is that it does not recover every loaded cell during the barcoding process and after the application of quality control filters. In our case, over 85% of cells freshly isolated from veins were viable, yet a lower-than-expected number of cells were recovered in each condition. We hypothesize that the low recovery rate of loaded cells is due to low RNA content cells existing in the vein wall or potentially fracture of myelin (found within the adventitia) into fragments during tissue digestion, leading to an artificially high initial cell count. Another limitation is related to the IVC ligation model. In human, DVT may occur in more distal veins (femoral and tibial) or more proximal, such as the iliofemoral or cava. It is possible that the pathophysiology revealed by the IVC ligation model may not be fully applicable to distal veins due to the heterogeneity between different vein segments. In humans, it is unclear exactly what causes DVT, although numerous risk factors are defined. Balancing the need to fill the knowledge gap of understanding the DVT response at the single-cell level, and the observation that human specimens of DVT and DVT tissue are similar in appearance to the mouse over time, we felt this model was best for this study^46,47^. Despite of its flaws, the IVC ligation model offers several features including the large vein size, high thrombosis incidence, and consistent thrombus weight/length that are particularly suitable for investigating single-cell transcriptomic changes in the vein wall in response to DVT. Finally, it would be more informative to conduct scRNA-seq analysis on human DVT-bearing vein wall specimens. However, obtaining human DVT-adjacent vein wall specimens is exceedingly challenging. DVT is rarely treated with open surgery, as endovascular therapies are typically first line when invasive intervention is required. When open surgery is performed, surgery typically involves thrombectomy alone without vein removal. Future studies comparing this scRNA-seq dataset with one generated using an alternative murine model of DVT or across later timepoints would be of great interest.

In conclusion, for the first time we uncovered the cellular heterogeneity of murine IVC and mapped the molecular signatures of the DVT-bearing vein wall at the single-cell level. Our data provides unique insights into the transcriptional changes that occur within the vein wall during early DVT, which may contribute not only to thrombogenesis but also long-term adverse vein wall remodeling. This unbiased, granular analysis of the vein wall broadens our understanding of DVT, and may ultimately facilitate the development of effective therapeutics for DVT patients.

## Methods

### Mice

All animal studies were performed with the approval of the Institute Animal Care and Use Committee at the University of Wisconsin—Madison (Protocol #M005792). 8 to 12-week-old, weight-matched male C57BL/6 J mice (Jackson Laboratory, Stock #000664) were used for all studies, as the gonadal vein anatomy of female mice increases variability in DVT models^24^.

### IVC ligation mouse model of DVT

IVC ligation surgery was performed as previously described^43,48,49^. Mice were anesthetized with 5% isoflurane, and anesthesia was maintained using 2.5% isoflurane thereafter. 0.6 mg/kg sustained-release buprenorphine was administered subcutaneously before surgery. A midline laparotomy was performed and the IVC was exposed after performing a right medial visceral rotation. Back branches draining into the IVC between the renal and iliac veins were cauterized. Side branches were ligated with 7/0 polypropylene suture. A small window was made between the aorta and IVC immediately inferior to the insertion of the left renal vein, and the IVC was ligated at this location using 7/0 polypropylene suture. Sham surgery involved vessel dissection without interruption. The intra-abdominal contents were returned and the abdomen was closed in a layered fashion.

For single-cell studies, 24 h after mice underwent IVC ligation or sham surgery, mice were euthanized and perfused with PBS. Infrarenal IVCs were carefully separated from the adjacent tissue (aorta, psoas muscle, lymph nodes, adipose tissue, intraluminal thrombus) and collected for scRNA-seq.

For histologic analysis, mice underwent IVC ligation or sham surgery were euthanized 24 or 48 h postsurgery. IVC/thrombus was collected en-bloc, embedded in optimal cutting temperature compound, and cut into 6μm cross-sections for immunofluorescent staining.

### Sample preparation and sequencing

Mouse infrarenal IVCs were collected and sequentially digested in two digestion buffers (PBS containing 200 U/ml collagenase I (SCR103, Sigma Aldrich), 0.05 U/ml elastase (E1250, Sigma Aldrich), 5 U/ml neutral protease (LS02111, Worthington), and 0.3 U/ml deoxyribonuclease I (M6101, Promega)^50^ for 20 min, followed by DMEM containing 5 mg/ml collagenase type II [C6885, Sigma-Aldrich] and 0.5 mg/ml elastase [LS002292, Worthington Biochemistry]) for 2.5 min at 37 °C). The tissue suspension was filtered with a 40μm cell strainer, then centrifuged at 500 g for 5 min. Cells were resuspended with PBS containing 0.04% BSA. Single-cell suspensions from 5 DVT-adjacent IVCs or 8 sham-IVCs were pooled together as one sample. 8000 cells per sample were loaded on a Chromium Controller (10x Genomics). The scRNA-seq libraries were constructed using the Chromium Single Cell 3′ v3.1 Reagent Kit according to the manufacturer’s guidelines (10x Genomics). cDNA libraries were uniquely sample indexed and pooled for sequencing. A MiSeq (Illumina) sequencing run was used to sample balance on a NovaSeq S1 flowcell (Illumina) using a 2×50 bp sequencing reaction targeting >90,000 reads/cell.

### Data preprocessing and cell clustering

Raw Illumina sequencing reads were aligned to mm10 (GENCODE vM23/Ensembl 98) reference genome and, subsequently, genes were quantified as UMI counts using Cell Ranger Count v6.1.2 pipeline (10x Genomics Cloud Analysis). Downstream analysis was performed on filtered feature counts generated by Cell Ranger using Seurat v4.1.0. Cells containing <800 genes or <1000 total number of RNA counts or >25% mitochondrial transcripts were considered as low-quality cells and removed from the dataset. Molecular count data from each sample were then normalized separately by performing sctransform(vst) and integrated into a single dataset by running sctransform-based workflows provided by R package Seurat^51^. After performing linear (PCA) and non-linear (umap) dimensional reduction as well as cell clustering on the integrated dataset, the cell type identity of each cluster was validated with a two-step approach. First, we utilized a Wilcoxon ranked sum in which the normalized gene expression values for one cell type were tested against normalized gene expression values for all other cell types. Resulting *p*-values were adjusted using the Bonferroni correction based on the enriched genes identified in each cluster (adjusted *p* < 0.0001, log2 fold change > 0.25). Enriched genes were then compared to previously identified marker genes to assign cell identity to each cell population. Secondly, we investigated the well-known canonical marker genes by plotting the distribution of expression values by cluster with violin plot and dot plot.

### Differential expression and pathway enrichment analysis

Differential expression analysis between DVT and sham groups was performed by utilizing R package MAST^52^. The whole differential expression analysis results were then used to generate volcano plot by using R package EnhancedVolcano (https://github.com/kevinblighe/EnhancedVolcano). The DEGs were a subset of the differential expression analysis results by applying the thresholds: log2 fold change > 0.25, Bonferroni adjusted *p*-value < 0.0001 and at least 10% of cells in either of the 2 groups express that gene. DEGs were subject to pathway enrichment analysis by utilizing R package clusterProfiler^53^.

### Cell–cell communication analysis using CellChat

We applied an established method CellChat^23^ to infer cell-cell communication across ECs, SMCs, fibroblasts, monocytes/macrophages, neutrophils, T/NK, and B cells. Clusters with less than 10 cells in either sham or DVT group were filtered out. The statistical significance of communication probability values was assessed using a permutation test. *P* < 0.05 was considered significant.

### Single cell weighted gene co-expression network analysis (scWGCNA)

R package scWGCNA (https://github.com/smorabit/scWGCNA) and WGCNA^54^ were utilized to describe the correlation patterns among genes and find the modules of highly correlated genes in SMCs. Before running the WGCNA pipeline, genes that are expressed in less than 5% of cells were excluded and transcriptionally similar cells were aggregated into pseudo-bulk metacells. In order to construct co-expression network and identify the co-expressed gene modules, 22, determined by calling the function TestSoftPowers, was selected as the soft-power threshold. Gene ontology enrichment analysis was performed on each module by using clusterProfiler. R package GeneOverlap (http://shenlab-sinai.github.io/shenlab-sinai/) was used to compare scWGCNA modules with SMC marker genes as well as the overlap between transcription factor target genes and co-expressed gene modules.

### Immunofluorescent staining

Tissue sections were fixed with 4% PFA, permeabilized with 0.1% Triton X-100, and blocked with 3% BSA. Sections were incubated at 4 °C overnight in 0.3% BSA containing primary antibodies: FITC anti-α-smooth muscle actin (ab8211, Abcam; 1:500), anti-Ly6G (127602, BioLegend; 1:800), anti-MYH11 (ab125884, Abcam, 1:200), anti-Fibronectin-1 (sc-271098, Santa Cruz, 1:100), anti-CD36 (AF2519, R&D systems, 1:100), anti-KLF2 (NBP2-45510, Novus Biologicals, 1:100), anti-ACTC1 (MAB93081-SP, R&D systems, 1:100), or anti-COL1A1 (NB600-408, Novus Biologicals, 1:100). After several washes with PBS, sections were incubated with Alexa Fluor 488- or Alexa Fluor 594-conjugated secondary antibodies for 1 h at room temperature. Negative control slides were stained with secondary antibody only. TUNEL staining was performed on fresh frozen sections using an In Situ Cell Death Detection Kit (Roche, Catalog #12 156 792 910) according to manufacture instructions. Co-staining with FITC anti-α-smooth muscle actin (ab8211, Abcam; 1:500) was performed. DAPI-containing mounting media (GBI Labs, Catalog #E19-100) was used as a counterstain. Images were acquired with a Nikon A1RS confocal microscope system.

### Statistics and reproducibility

In Supplementary Figure 1a, data were presented as mean ± SD, and visualized with GraphPad Prism 8 (GraphPad Software, Inc). *n* = 8 mice in sham group, *n* = 5 mice in DVT group. For histologic analysis, *n* = 3 mice in each group.

### Reporting summary

Further information on research design is available in the Nature Portfolio Reporting Summary linked to this article.

## Supplementary information

Supplementary Information

Description of Additional Supplementary Files

Supplementary Data 1

Supplementary Data 2

Supplementary Data 3

Supplementary Data 4

Supplementary Data 5

Supplementary Data 6

Supplementary Data 7

Reporting Summary

## Data Availability

scRNA-seq data are accessible in Gene Expression Omnibus under the accession number GSE221978. Source data underlying Figs. 1c, d, 2b, c, g–i, 3b, c, 4c, d, 5b, c, 6a, and d are presented in Supplementary Data 7. Other data are available from the corresponding authors on reasonable request.

## Code availability

The full code used in data processing are available in GitHub (https://github.com/hyang333/DVT_scRNAseq), 10.5281/zenodo.7523090.
