# Supplementary material for: A vein wall cell atlas of murine venous thrombosis determined by single-cell RNA sequencing
Source: Commun Biol. 2023 Jan 31;6:130. doi: 10.1038/s42003-023-04492-z (PMC9889765; doi:10.1038/s42003-023-04492-z)
Supplement: Supplementary file 1 — Supplementary Information [file 42003_2023_4492_MOESM1_ESM.pdf]

Supplementary Figure 1

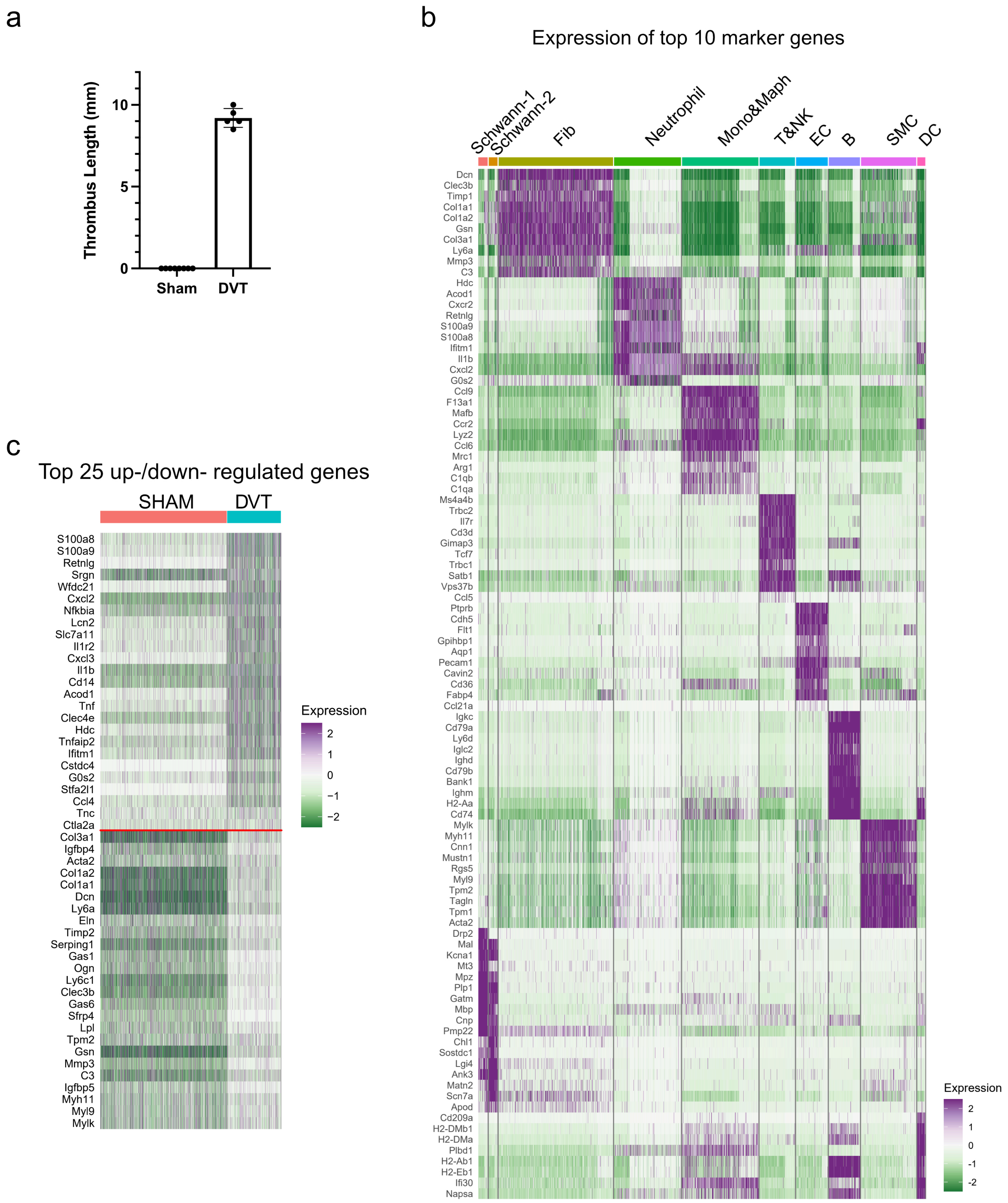

Supplementary Figure 1. Transcriptional differences between sham and deep vein thrombosis (DVT). (a) Thrombus length of sham and DVT groups 24 hours after inferior vena cava (IVC) ligation. n=8 mice in sham group, n=5 mice in DVT group. Data were presented as mean±SD. (b) Heatmap of the top 10 enriched genes in each cell population. (c) Heatmap of the top 25 up- and down- regulated genes in DVT compared to sham group. Up- and down- regulated genes were separate by a red line.

Supplementary Figure 2

a

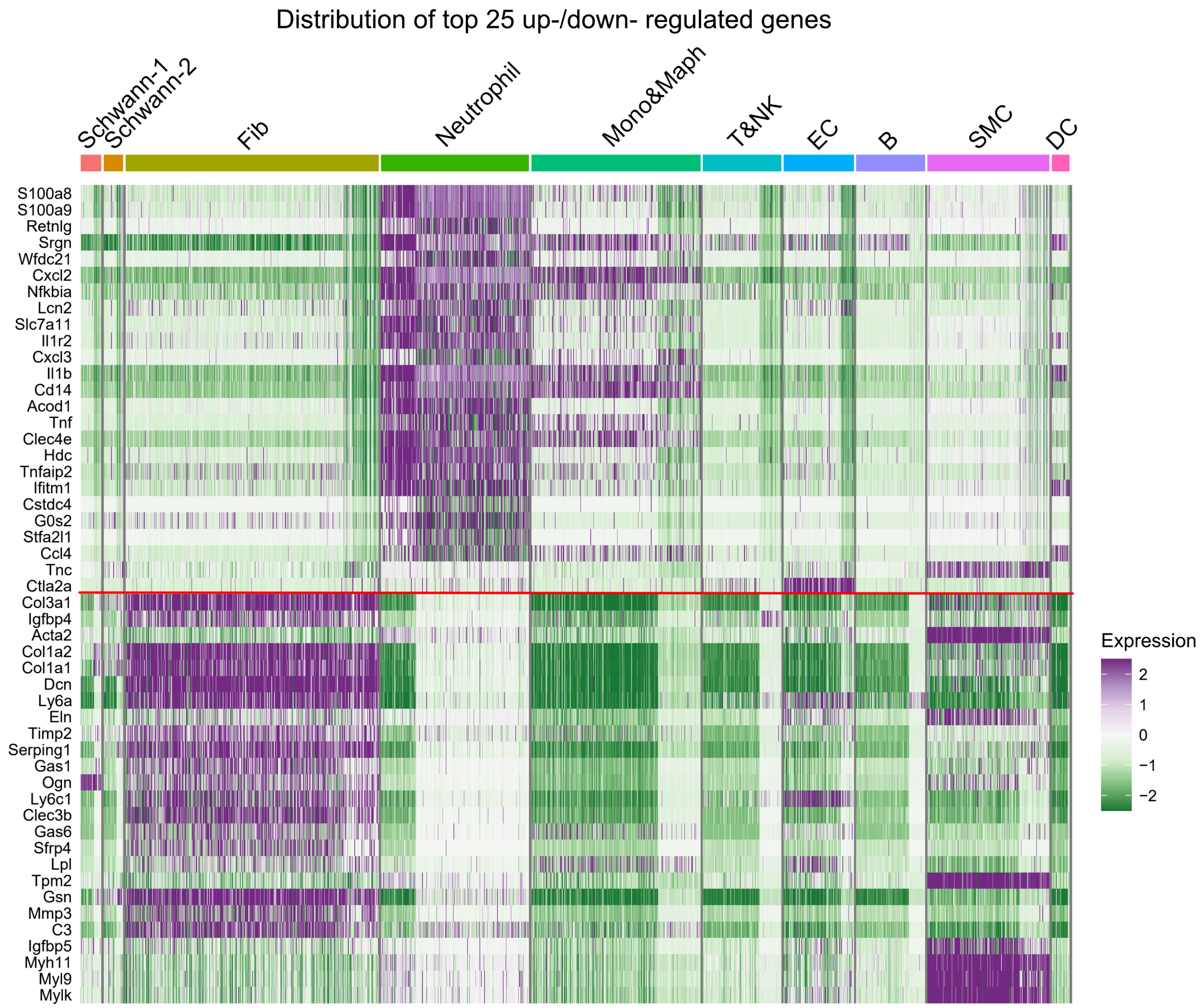

b

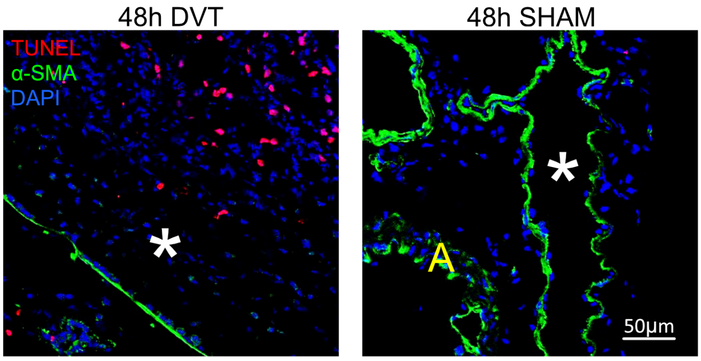

Supplementary Figure 2. Cellular and molecular changes induced by DVT. (a) Heatmap of the top 25 up- and down- regulated genes (DVT vs sham) in each cell population. Up- and down- regulated genes were separate by a red line. (b) TUNEL staining of IVC or IVC/thrombus cross-sections 48 hours after sham surgery or IVC ligation (DVT group). Red, TUNEL; green, smooth muscle cell marker α-smooth muscle actin; blue, DAPI. Vessel lumen or thrombus depicted with asterisk. Yellow “A” indicates aorta. N=3 mice for each group.

Supplementary Figure 3

a

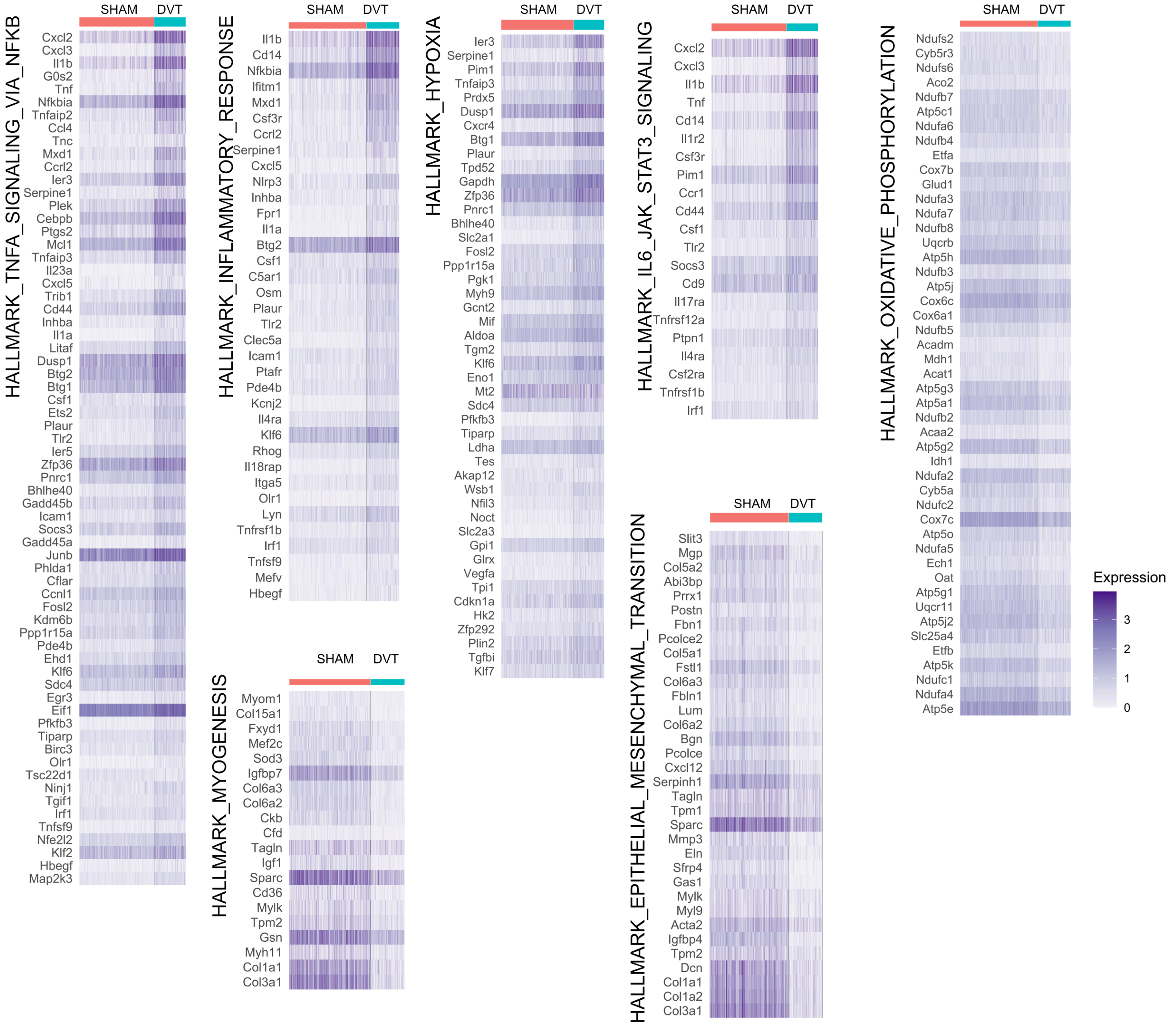

b

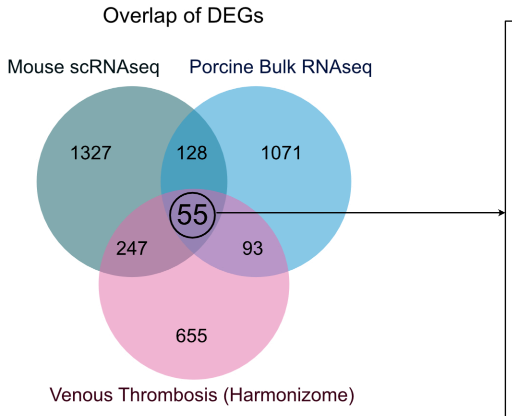

VCAM1	PLAT	LMCD1	WIPI1
TXNRD1	PFKFB3	JAK2	CSF1
TNFRSF1A	PDGFRA	ITGA5	CFLAR
TNFAIP3	PDE4B	IL1R1	CD44
TJP1	PCDH7	IGFBP4	BIRC3
STAT3	PARP1	HIF1A	BCL3
SPP1	ODC1	HBEGF	BACH1
SOD2	NGF	HADH	ATF4
SLC29A1	NFKB2	GAS6	ALOX5AP
SERPINE1	NCL	FOSL1	AKAP12
S100A10	MXI1	FGFR1	
RUNX1	MMP3	EMP3	
PTGS2	MAPKAPK2	EGFR	
PRNP	MAP2K3	DUSP5	
PRKACB	LPAR1	CXCL2	

Supplementary Figure 3. Altered genes in DVT compared to the sham group. (a) Heatmap of gene sets altered the most from Figure 1g in sham and DVT groups. (b) Venn diagram of differentially expressed genes (DEGs) from single-cell RNAseq of mouse DVT, as well as published DEGs from bulk RNAseq of a porcine DVT model, and DEGs inferred to be associated with human DVT from the Comparative Toxicogenomics Database.

Supplementary Figure 4

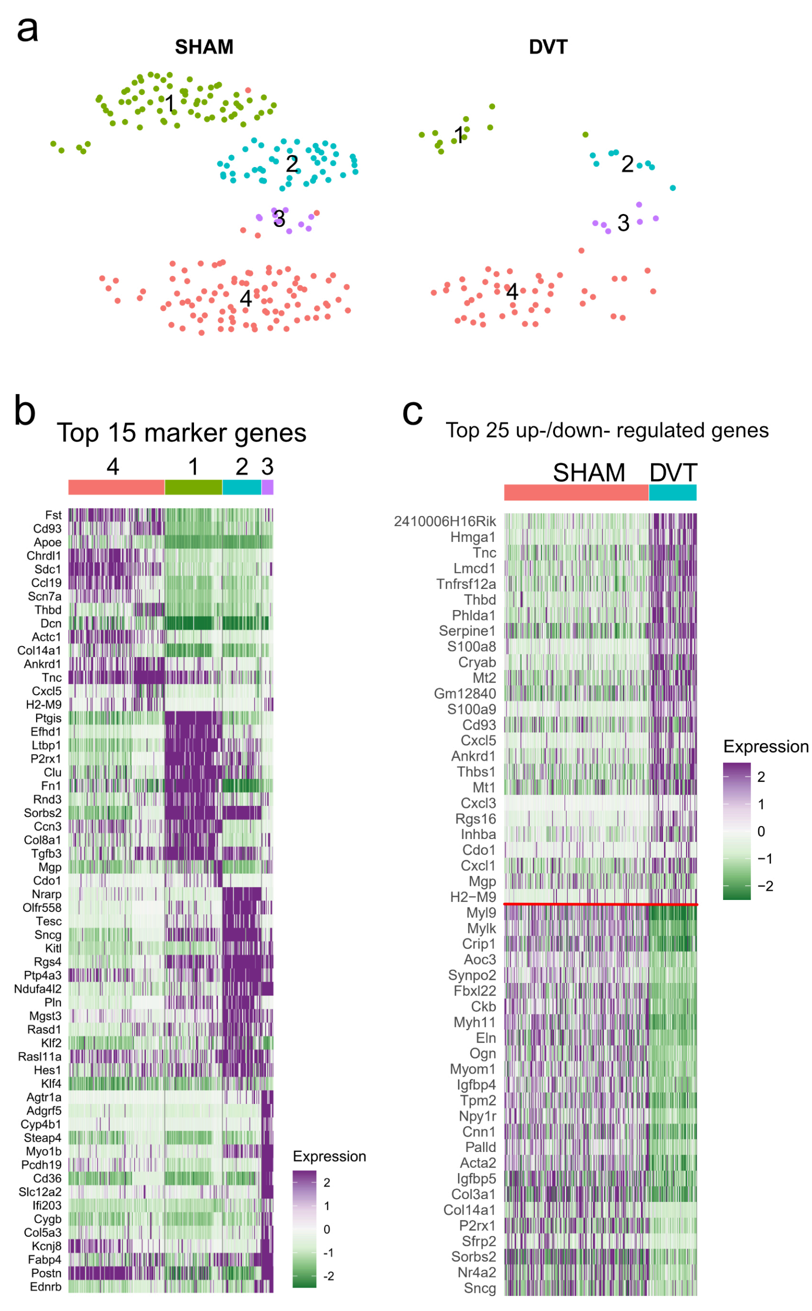

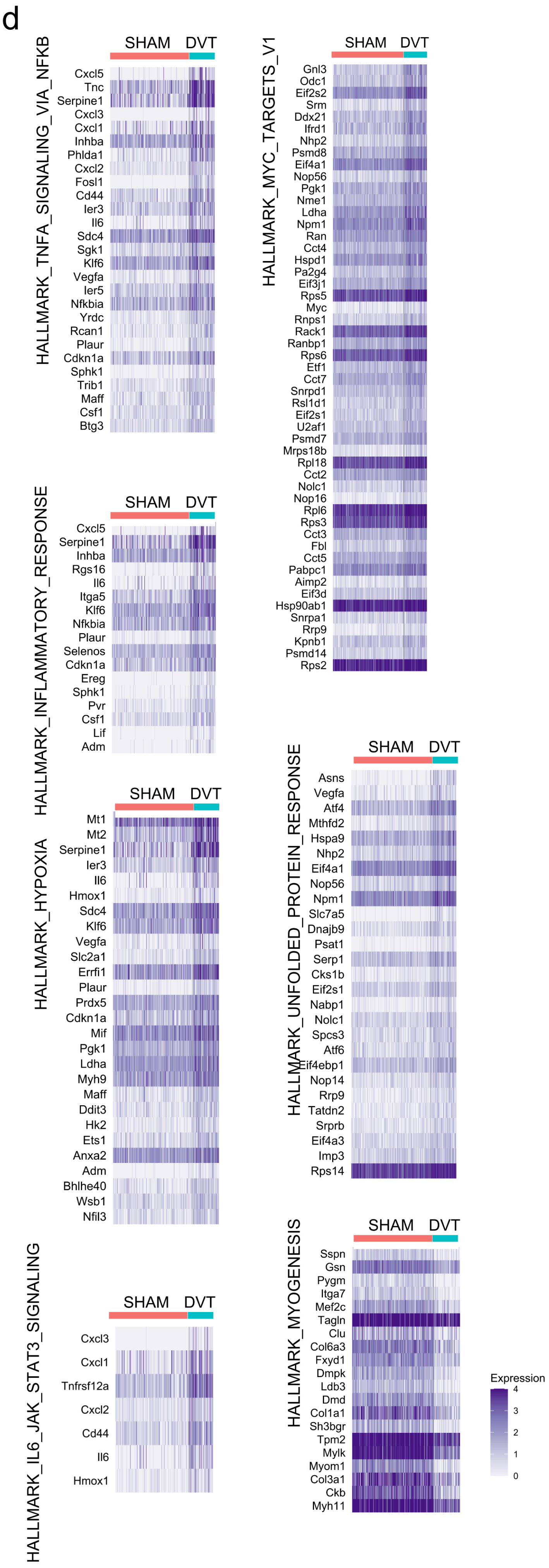

Supplementary Figure 4. Transcriptional profile of smooth muscle cell (SMC) sub-populations. (a) UMAP plot of SMC sub-populations in sham and DVT groups. (b) Heatmap of the top 15 enriched genes in each SMC sub-population in comparison to the other three sub-populations. (c) Heatmap of the top 25 up- and down- regulated genes in DVT compared to sham group. Up- and down- regulated genes were separate by a red line. (d) Heatmap of gene sets altered the most from Figure 2e in sham and DVT groups.

Supplementary Figure 5

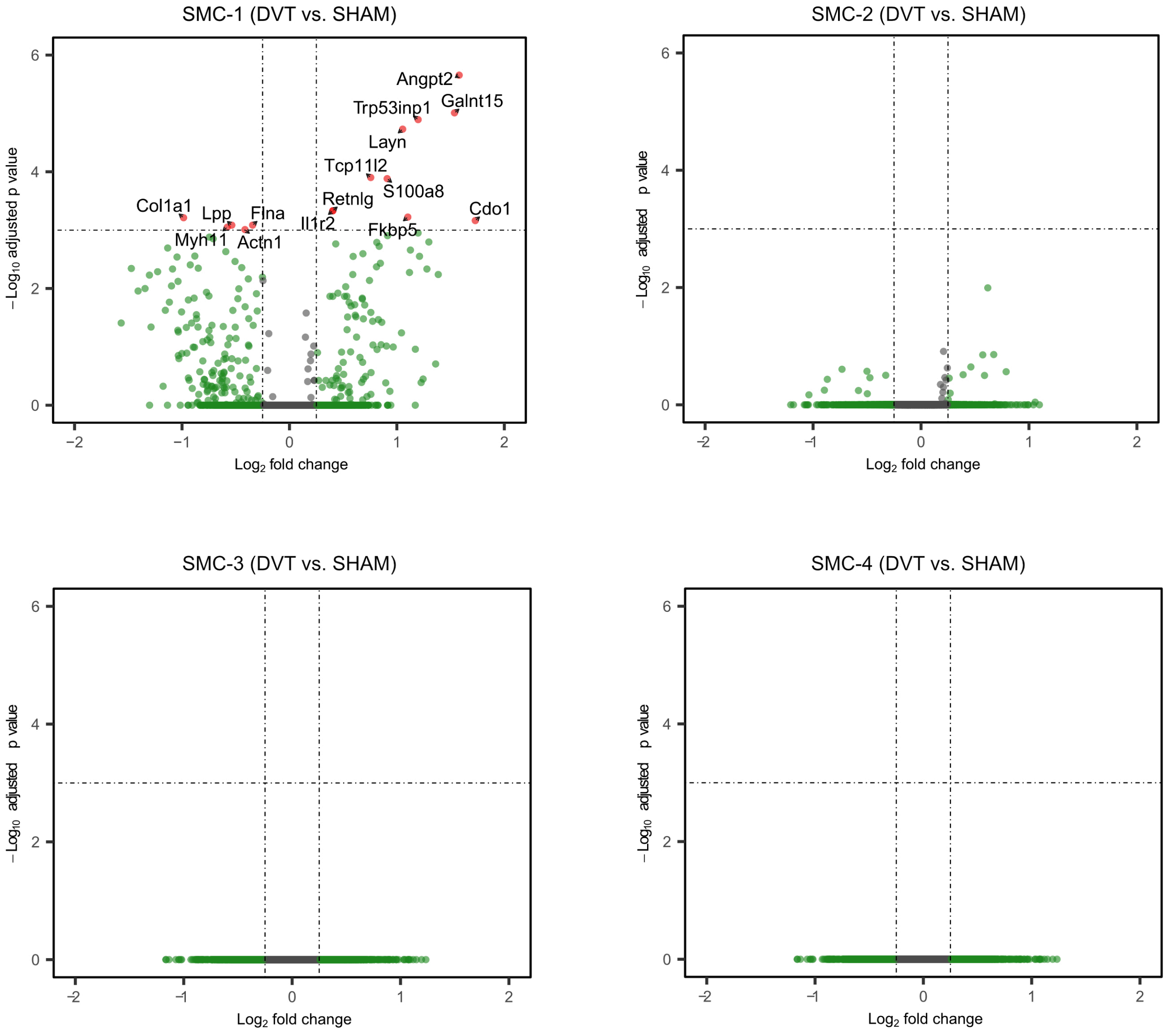

Supplementary Figure 5. Volcano plot of DEGs in each SMC sub-population (DVT vs sham group).

Supplementary Figure 6

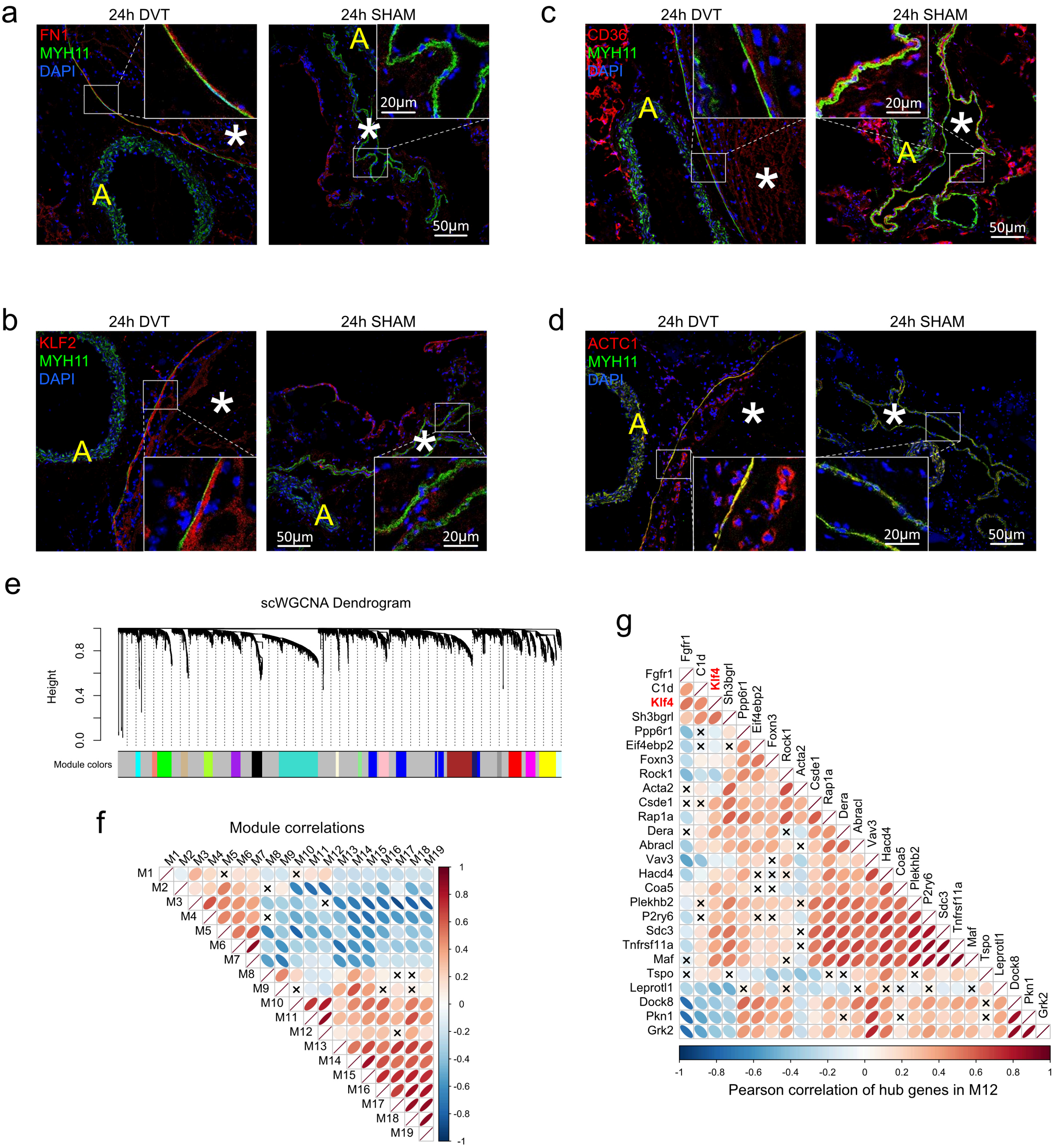

Supplementary Figure 6. Characterization of SMC sub-populations. (a-d) Co-staining of SMC marker, smooth muscle myosin heavy chain 11 (MYH11), and marker genes of each SMC sub-population (Fibronectin-1 [FN1] for SMC-1 [a], Krüppel-like factor 2 [KLF2] for SMC-2 [b], CD36 for SMC-3 [c], and actin alpha cardiac muscle 1 [ACTC1] for SMC-4 [d]) within IVC or IVC/thrombus cross-sections 24 hours after sham surgery or IVC ligation (DVT group). DAPI was used to stain nuclei. Vessel lumen or thrombus depicted with asterisk. Yellow “A” indicates aorta. Area encompassed by white box is shown magnified in the insert. N=3 mice for each group. (e) Dendrogram of single-cell weighted gene co-expression network analysis (scWGCNA) in total SMCs of sham and DVT data combined. (f) Correlation analysis of each co-expressed gene module with other modules. (g) Pearson correlation analysis of hub genes in module 12.

Supplementary Figure 7

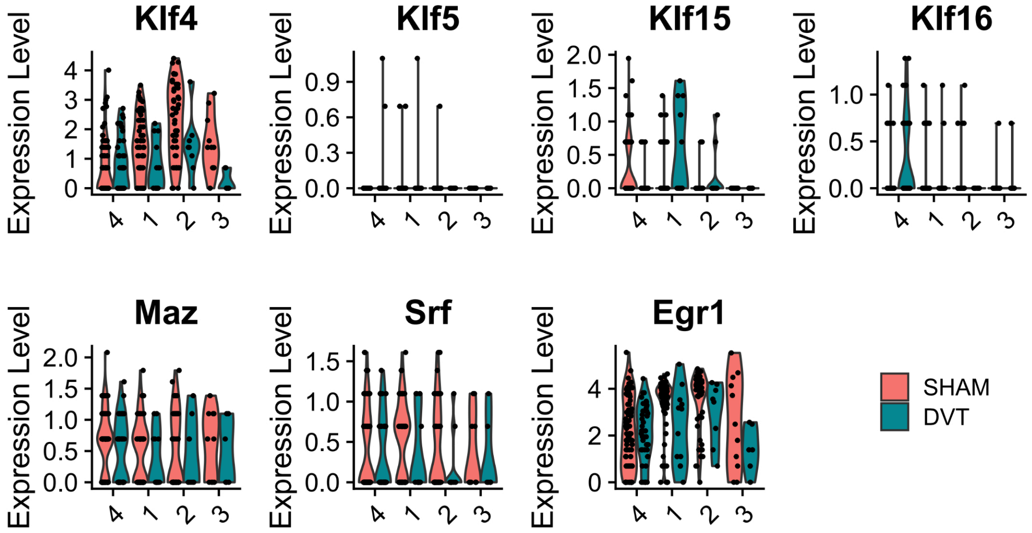

Supplementary Figure 7. Expression of transcription factors identified in Figure 2i within each SMC sub-population in the sham and DVT conditions.

Supplementary Figure 8

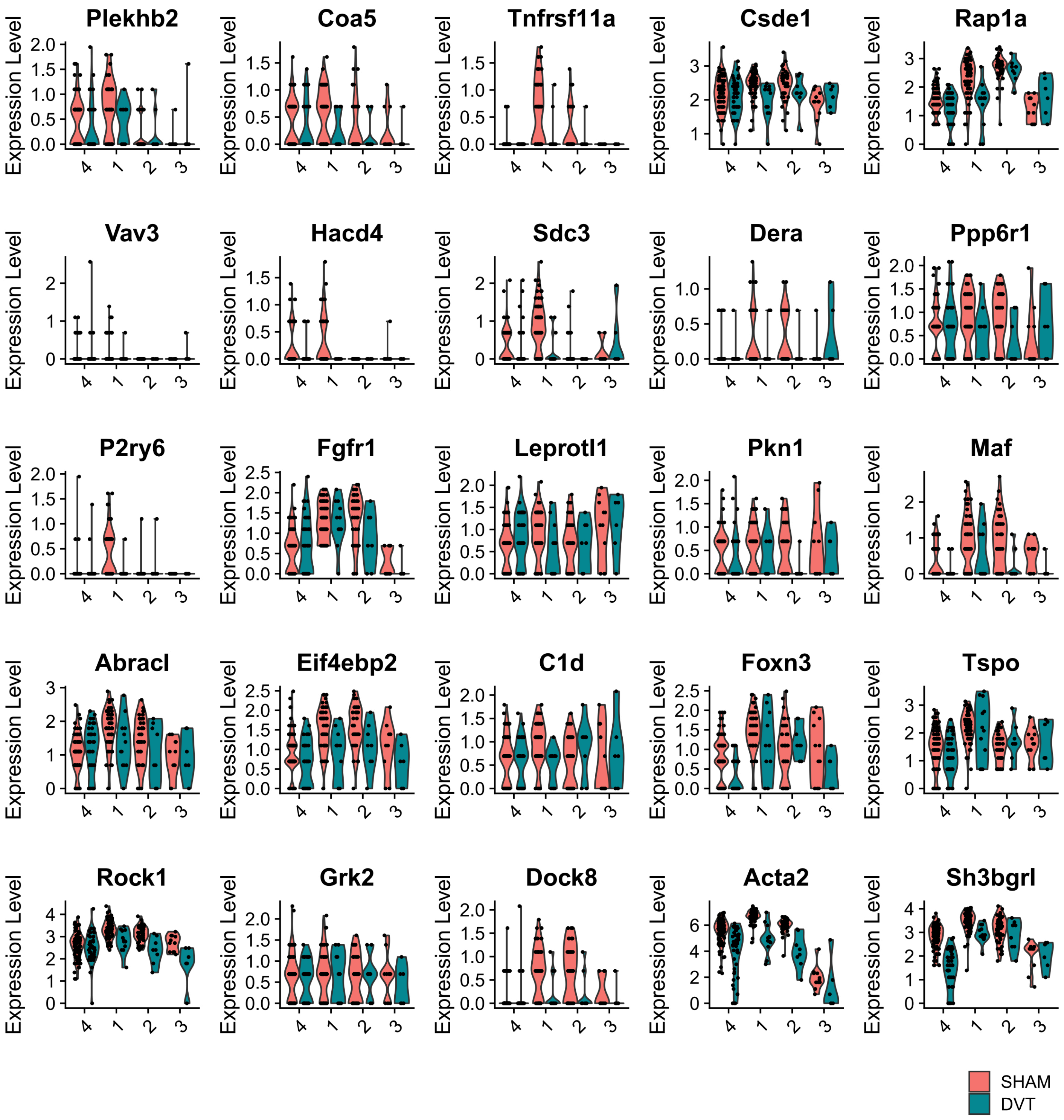

Supplementary Figure 8. Module 12 hub gene expression patterns within each SMC sub-population in the sham and DVT conditions.

Supplementary Figure 9

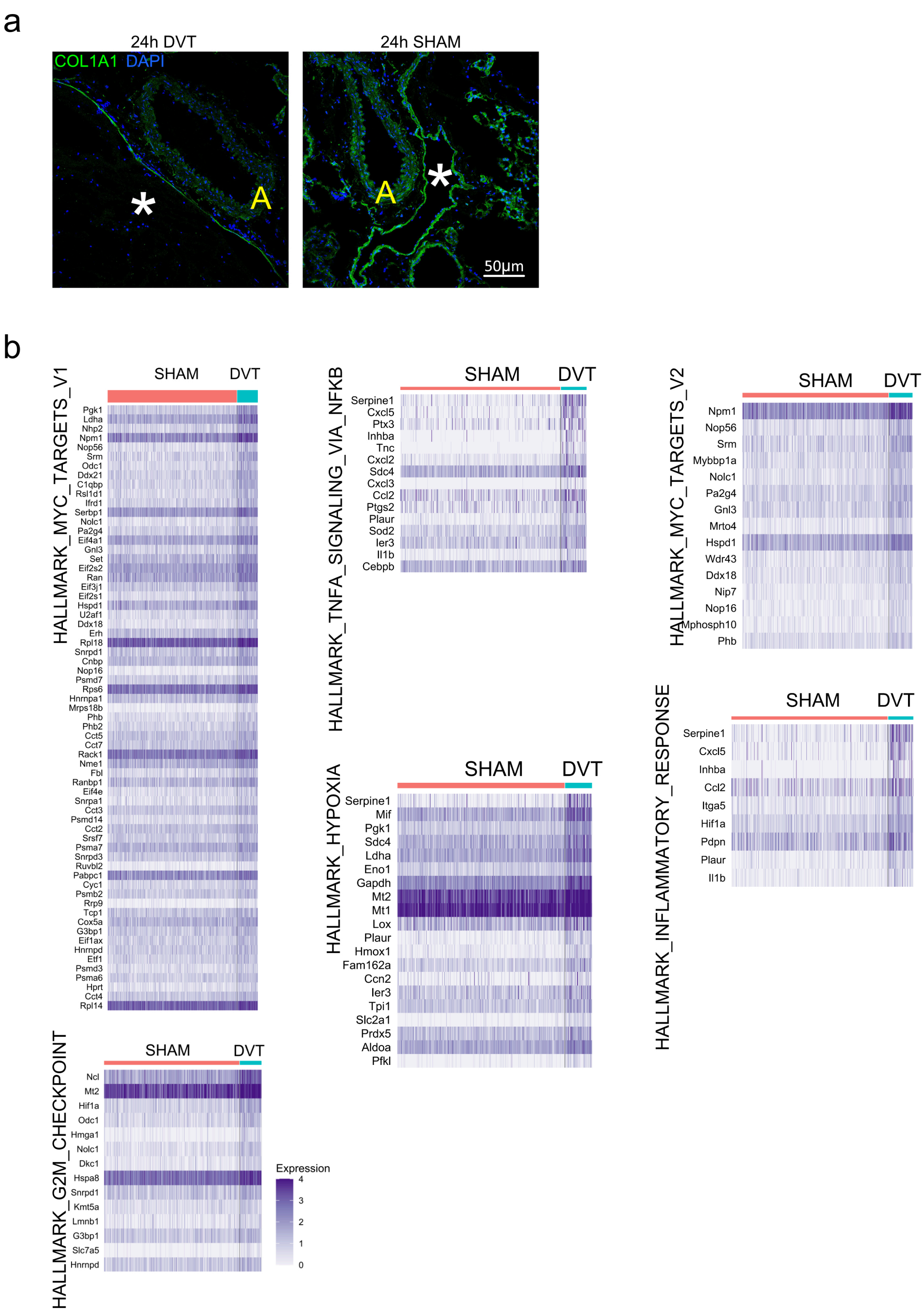

Supplementary Figure 9. Gene expression profile of fibroblasts. (a) Immunostaining of COL1A1 within IVC or IVC/thrombus cross-sections 24 hours after sham surgery or IVC ligation (DVT group). DAPI was used to stain nuclei. Vessel lumen or thrombus depicted with asterisk. Yellow "A" indicates aorta. N=3 mice for each group. (b) Heatmap of gene sets altered the most from Figure 3g in sham and DVT groups.

Supplementary Figure 10

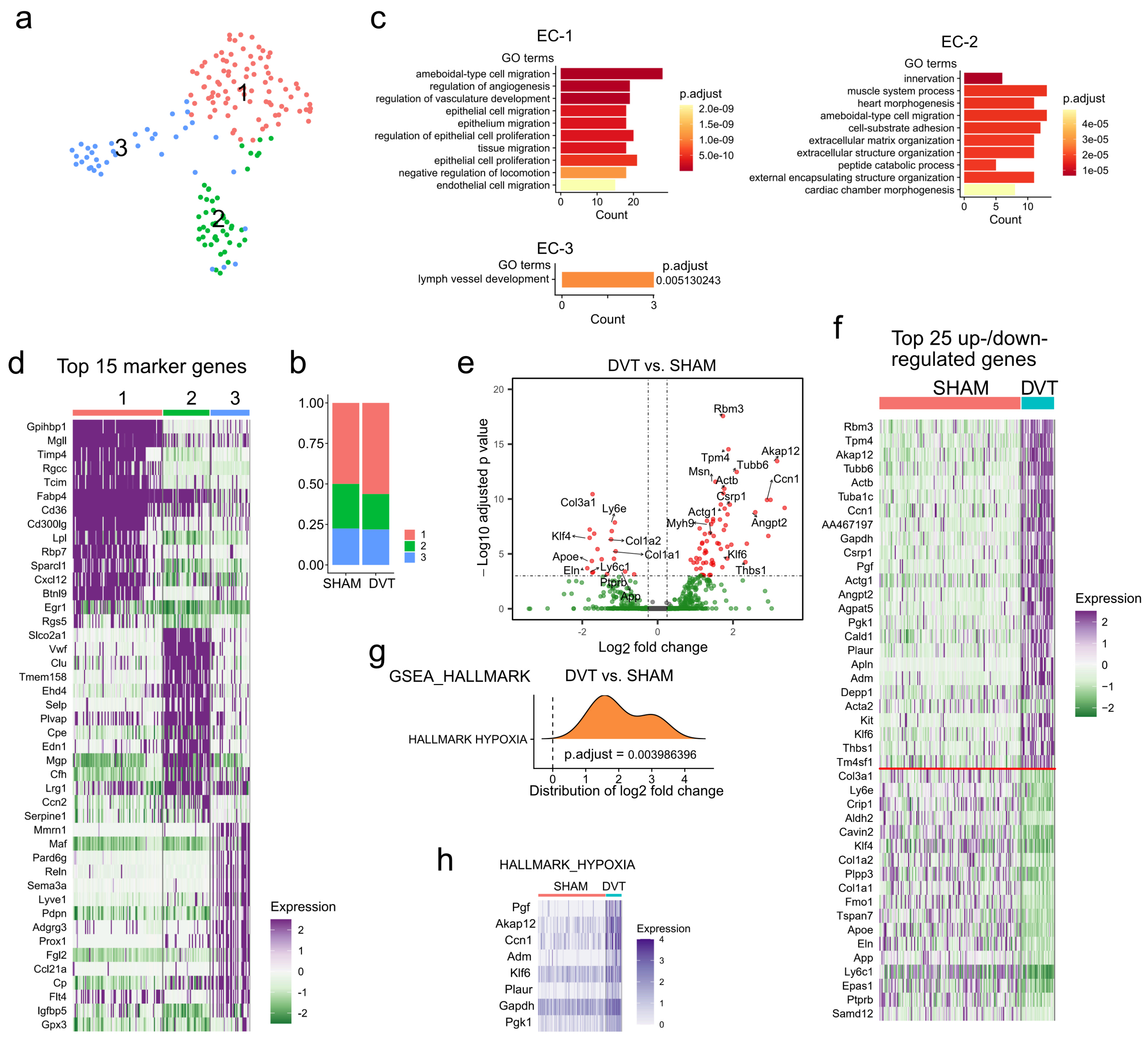

Supplementary Figure 10. Gene expression heterogeneity of endothelial cells (ECs). (a) UMAP plot of sub-populations in ECs of sham and DVT combined. (b) Relative distribution of sub-populations in sham and DVT groups. (c) Gene Ontology (GO) analysis of each EC sub-population in comparison to the other two sub-populations. (d) Heatmap of the top 15 enriched genes in each EC sub-population in comparison to the other two sub-populations. (e) Volcano plot of DEGs in total ECs (DVT vs sham group). (f) Heatmap of the top 25 up- and down- regulated genes in DVT compared to sham group. Up- and down- regulated genes were separate by a red line. (g) GSEA of the altered gene set in DVT versus sham group. (h) Heatmap of the altered gene set from (g) in sham and DVT groups.

Supplementary Figure 11

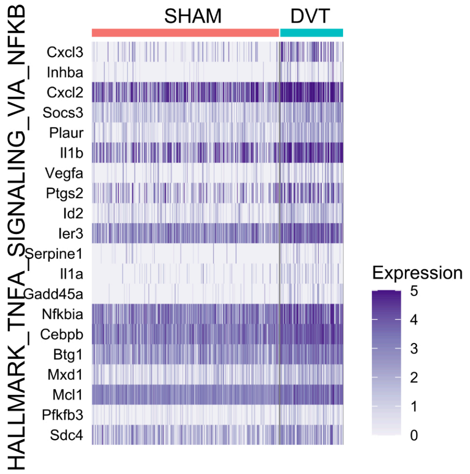

Supplementary Figure 11. Heatmap of gene sets altered the most from Figure 5g in sham and DVT groups.

Supplementary Figure 12

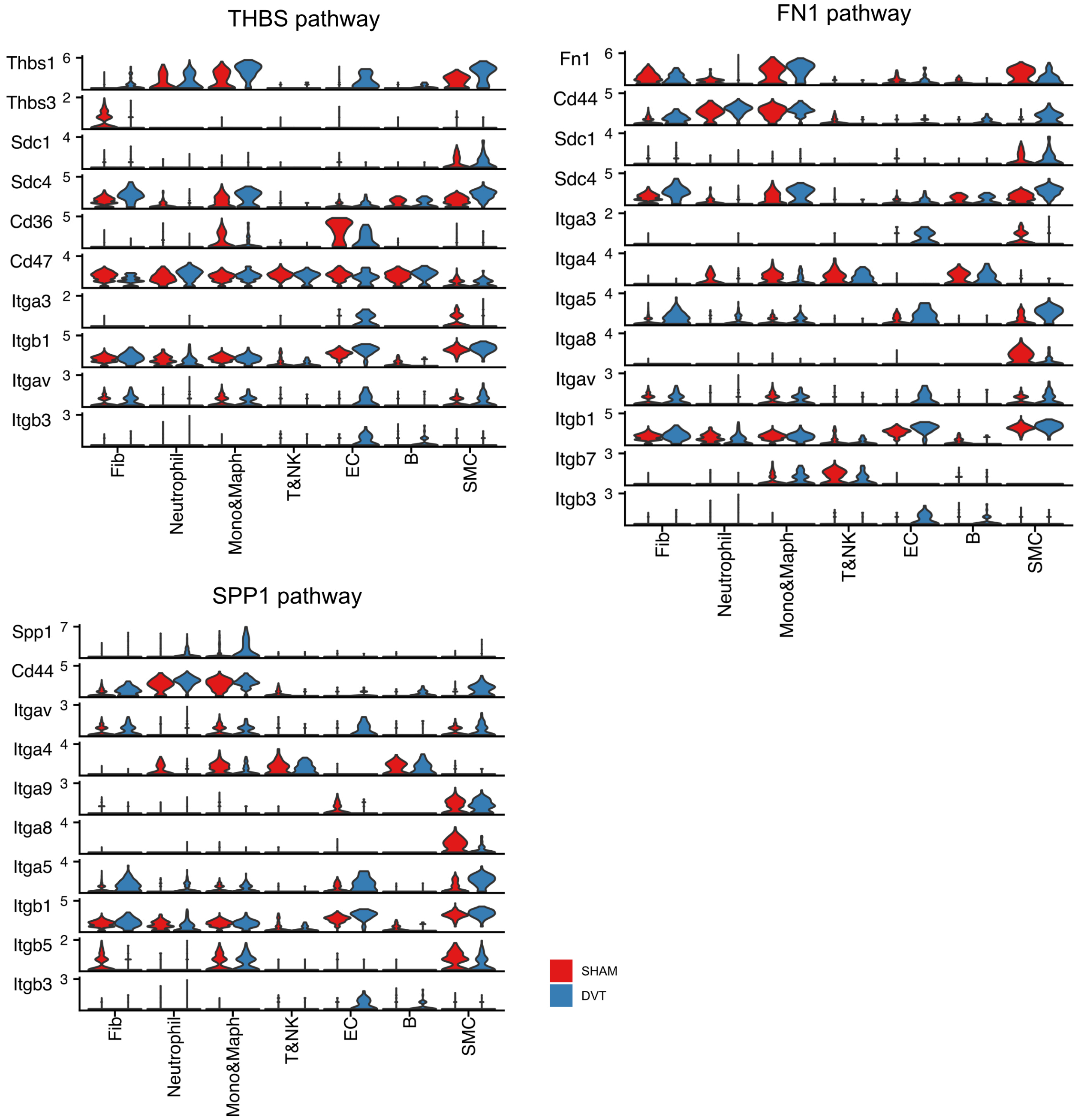

Supplementary Figure 12. Gene expression distribution of genes in THBS, SPP1, and FN1 signaling pathways in sham and DVT groups.
