# Supplementary material for: A vein wall cell atlas of murine venous thrombosis determined by single-cell RNA sequencing
Source: Commun Biol. 2023 Jan 31;6:130. doi: 10.1038/s42003-023-04492-z (PMC9889765; doi:10.1038/s42003-023-04492-z)
Supplement: Supplementary file 2 — Description of Additional Supplementary Files [file 42003_2023_4492_MOESM2_ESM.pdf]

Description of Additional Supplementary Files

File name: Supplementary Data 1

Description: Total differentially expressed genes in DVT versus sham group

File name: Supplementary Data 2

Description: Differentially expressed genes in DVT versus sham group (smooth muscle cells)

File name: Supplementary Data 3

Description: Differentially expressed genes in DVT versus sham group (fibroblasts)

File name: Supplementary Data 4

Description: Differentially expressed genes in DVT versus sham group (endothelial cells)

File name: Supplementary Data 5

Description: Differentially expressed genes in DVT versus sham group (neutrophils)

File name: Supplementary Data 6

Description: Differentially expressed genes in DVT versus sham group (monocytes and macrophages)

File name: Supplementary Data 7

Description: Source data for Fig 1c-d, 2b-c, 2g-i, 3b-c, 4c-d, 5b-c, 6a, and 6d
