# Supplementary material for: A vein wall cell atlas of murine venous thrombosis determined by single-cell RNA sequencing
Source: Commun Biol. 2023 Jan 31;6:130. doi: 10.1038/s42003-023-04492-z (PMC9889765; doi:10.1038/s42003-023-04492-z)
Supplement: Supplementary file 10 — Reporting Summary [file 42003_2023_4492_MOESM10_ESM.pdf]

nature portfolio

Corresponding author(s): Ting Zhou and Bo Liu

Last updated by author(s): Dec 30, 2022

Reporting Summary

Nature Portfolio wishes to improve the reproducibility of the work that we publish. This form provides structure for consistency and transparency in reporting. For further information on Nature Portfolio policies, see our Editorial Policies and the Editorial Policy Checklist.

Statistics

For all statistical analyses, confirm that the following items are present in the figure legend, table legend, main text, or Methods section.

n/a	Confirmed	
☐	☒	The exact sample size (*n*) for each experimental group/condition, given as a discrete number and unit of measurement
☐	☒	A statement on whether measurements were taken from distinct samples or whether the same sample was measured repeatedly
☐	☒	The statistical test(s) used AND whether they are one- or two-sided<br>*Only common tests should be described solely by name; describe more complex techniques in the Methods section.*
☒	☐	A description of all covariates tested
☐	☒	A description of any assumptions or corrections, such as tests of normality and adjustment for multiple comparisons
☒	☐	A full description of the statistical parameters including central tendency (e.g. means) or other basic estimates (e.g. regression coefficient) AND variation (e.g. standard deviation) or associated estimates of uncertainty (e.g. confidence intervals)
☐	☒	For null hypothesis testing, the test statistic (e.g. *F*, *t*, *r*) with confidence intervals, effect sizes, degrees of freedom and *P* value noted<br>*Give P values as exact values whenever suitable.*
☒	☐	For Bayesian analysis, information on the choice of priors and Markov chain Monte Carlo settings
☒	☐	For hierarchical and complex designs, identification of the appropriate level for tests and full reporting of outcomes
☒	☐	Estimates of effect sizes (e.g. Cohen's *d*, Pearson's *r*), indicating how they were calculated

Our web collection on statistics for biologists contains articles on many of the points above.

Software and code

Policy information about availability of computer code

Data collection	ScRNA-seq data are accessible in Gene Expression Omnibus under the accession number GSE221978. Other data are available from the corresponding authors on reasonable request.
Data analysis	The full code used in data processing are available in GitHub (https://github.com/hyang333/DVT_scRNAseq).

For manuscripts utilizing custom algorithms or software that are central to the research but not yet described in published literature, software must be made available to editors and reviewers. We strongly encourage code deposition in a community repository (e.g. GitHub). See the Nature Portfolio guidelines for submitting code & software for further information.

Data

Policy information about availability of data

All manuscripts must include a data availability statement. This statement should provide the following information, where applicable:

- Accession codes, unique identifiers, or web links for publicly available datasets
- A description of any restrictions on data availability
- For clinical datasets or third party data, please ensure that the statement adheres to our policy

Data Availability: ScRNA-seq data are accessible in Gene Expression Omnibus under the accession number GSE221978. Other data are available from the corresponding authors on reasonable request.
Code availability: The full code used in data processing are available in GitHub (https://github.com/hyang333/DVT_scRNAseq).

Human research participants

Policy information about studies involving human research participants and Sex and Gender in Research.

Reporting on sex and gender	N/A
Population characteristics	N/A
Recruitment	N/A
Ethics oversight	N/A

Note that full information on the approval of the study protocol must also be provided in the manuscript.

Field-specific reporting

Please select the one below that is the best fit for your research. If you are not sure, read the appropriate sections before making your selection.

☒ Life sciences ☐ Behavioural & social sciences ☐ Ecological, evolutionary & environmental sciences

For a reference copy of the document with all sections, see nature.com/documents/nr-reporting-summary-flat.pdf

Life sciences study design

All studies must disclose on these points even when the disclosure is negative.

Sample size	5 DVT-adjacent IVCs or 8 sham-IVCs were pooled to generate single cell suspension.
Data exclusions	Cells containing <800 genes or <1000 total number of RNA counts or >25% mitochondrial transcripts were considered as low quality cells and removed from the dataset.
Replication	N/A
Randomization	N/A
Blinding	DVT inducing surgery, single cell suspension preparation, and data analysis was conducted by different researchers.

Reporting for specific materials, systems and methods

We require information from authors about some types of materials, experimental systems and methods used in many studies. Here, indicate whether each material, system or method listed is relevant to your study. If you are not sure if a list item applies to your research, read the appropriate section before selecting a response.

Materials & experimental systems

n/a	Involved in the study	
☐	☒	Antibodies
☒	☐	Eukaryotic cell lines
☒	☐	Palaeontology and archaeology
☐	☒	Animals and other organisms
☒	☐	Clinical data
☒	☐	Dual use research of concern

Methods

n/a	Involved in the study	
☒	☐	ChIP-seq
☒	☐	Flow cytometry
☒	☐	MRI-based neuroimaging

Antibodies

Antibodies used	FITC anti-alpha-smooth muscle actin (ab8211, Abcam; 1:500), anti-Ly6G (127602, BioLegend; 1:800), anti-MYH11 (ab125884, Abcam, 1:200), anti-Fibronectin-1 (sc-271098, Santa Cruz, 1:100), anti-CD36 (AF2519, R&D systems, 1:100), anti-KLF2 (NBP2-45510, Novus Biologicals, 1:100), anti-ACTC1 (MAB93081-SP, R&D systems, 1:100), and anti-COL1A1 (NB600-408, Novus Biologicals, 1:100).
Validation	Antibodies were validated by the manufacturers.

Animals and other research organisms

Policy information about studies involving animals; ARRIVE guidelines recommended for reporting animal research, and Sex and Gender in Research

Laboratory animals	Mouse, C57BL6/J, male, age 8 weeks
Wild animals	N/A
Reporting on sex	8 week old, weight-matched male C57BL/6J mice (Jackson Laboratory, Stock #000664) were used for all studies, as the gonadal vein anatomy of female mice increases variability in DVT models
Field-collected samples	N/A
Ethics oversight	All animal studies were performed with the approval of the Institute Animal Care and Use Committee at the University of Wisconsin – Madison (Protocol #M005792).

Note that full information on the approval of the study protocol must also be provided in the manuscript.
